# NMR and HPLC profiling of bee pollen products from different countries

**DOI:** 10.1016/j.fochms.2022.100119

**Published:** 2022-07-06

**Authors:** Peng Lu, Saki Takiguchi, Yuka Honda, Yi Lu, Taichi Mitsui, Shingo Kato, Rina Kodera, Kazuo Furihata, Mimin Zhang, Ken Okamoto, Hideaki Itoh, Michio Suzuki, Hiroyuki Kono, Koji Nagata

**Affiliations:** a Department of Applied Biological Chemistry, Graduate School of Agricultural and Life Science, The University of Tokyo, 1-1-1 Yayoi, Bunkyo-ku, Tokyo 113-8657, Japan; b Nagaragawa Research Center, API Co., Ltd., 692-3 Nagara, Gifu-City, Gifu 502-0071, Japan; c Advanced Instrumental Analysis Unit, Graduate School of Agricultural and Life Science, The University of Tokyo, 1-1-1 Yayoi, Bunkyo-ku, Tokyo 113-8657, Japan; d Agricultural Bioinformatics Research Unit, Graduate School of Agricultural and Life Science, The University of Tokyo, 1-1-1 Yayoi, Bunkyo-ku, Tokyo 113-8657, Japan

**Keywords:** Bee pollen, Component profiling, Metabolomics, Flavonoids, Principal component analysis

## Abstract

Bee pollen, a beehive product collected from flowers by honeybees, contains over 250 biological substances, and has attracted increasing attention as a functional food. However, commercial bee pollen products are often multifloral, and samples from different countries vary significantly. There is no universal standard for objective quality assessment of bee pollen based on its chemical composition. Here, we report metabolomic analysis of 11 bee pollen samples from Spain, China, and Australia for quality control. The characteristics of the samples depend on the sucrose, nucleoside, amino acid, and flavanol concentrations. Bee pollen samples from Spain and Australia had higher sucrose and adenosine concentrations, whereas those from China had higher trigonelline, uridine, and cytidine concentrations. Interestingly, acetic acid was only detected in samples from China. These components can be used to identify the country of origin. The obtained profiles of the samples will contribute to universal standard development for bee pollen products.

## Highlights

• Analysis using NMR and HPLC can be used to identify countries of origin but not producing years of bee pollens.
• Flavanoids in bee pollens from different countries were characterized using HPLC.
• Bee pollens from Spain and Australia were high in sucrose and adenosine.
• Bee pollens from China were high in trigonelline, uridine, and cytidine.
• Only the bee pollens from China contained acetic acid.

## 1 Introduction

Bee pollen contains many essential amino acids needed for humans and has been consumed by humans as a valuable source of nutrients since ancient times (Linskens and Jorde, 1997, Pascoal et al., 2014). Several studies have reported that bee pollen contains a large number of compounds with health-promoting effects, including essential amino acids, antioxidants, vitamins, lipids, and other bioactive compounds (Ares et al., 2018, Feas et al., 2012). Bee pollen contains over 250 biological substances (Kieliszek et al., 2018), including 22 different amino acids, together with minor components including minerals (Zn, Cu, Fe, and a high K/Na ratio), nucleic acids, enzymes, phenolic compounds, and pigments (chlorophylls and carotenoids) (Campos et al., 2010, Rocchetti et al., 2019). Therefore, among the various beehive products, bee pollen has attracted increasing attention for human consumption (Kieliszek et al., 2018, Mărgăoan et al., 2019).

Bee pollen has been a special food across continents since ancient times. Ancient Egyptians and Greeks recognized the health benefits of bee pollen. Ancient Roman soldiers as well as native Americans carried bee pollen to provide sustenance and energy on long journeys. The ancient cultures of China and India have the same references to the health benefits of bee pollen. In New Zealand, the Māori have a long tradition of using bee pollen for food. After early civilizations identified its nutritional power, bee pollen is once again becoming an important part of the human diet in these decades. Due to its high nutritional profile, bee pollen is a popular food to athletes to enhance their athletic performance. In Japan, bee pollen has been imported for over 30 years and has become known as a health food. Researchers have analyzed its constituents to comprehensively map its nutritional benefits (Costa et al., 2019, Gonçalves et al., 2018, Xue et al., 2012, Zhang et al., 2022, Zhang et al., 2022).

In the past two decades, many studies have analyzed the components and biological activities of bee pollen and have shown its beneficial effects on the human immune defense system, along with many other activities (Mărgăoan et al., 2019). In addition, the consumption of pollen might reduce the risk of chronic diseases such as cancer, as well as cardiovascular and neurodegenerative diseases (Cornara et al., 2017, Denisow and Denisow-Pietrzyk, 2016, Morais et al., 2011, Rzepecka-Stojko et al., 2015). The actual phytochemical profile of bee pollen profile depends on its botanical origin and other factors such as soil type, beekeeping management practices, and climatic and preservation conditions (Almeida-Muradian et al., 2005, Komosinska-Vassev et al., 2015). However, bee pollen samples originating from different countries vary greatly, and there is no universal standard for objective evaluation of the qualities of bee pollen based on its chemical composition. In addition, commercially available bee pollen products are often multifloral (*i.e.*, formed from multiple plant sources), and plant sources can vary greatly due to various factors, even among bee pollen products produced in the same country (Feas et al., 2012). In some cases, the variation in the pollen source can affect the composition of the final product, which represents a quality control issue when selling bee pollen as a functional food (Denisow & Denisow-Pietrzyk, 2016).

High-performance liquid chromatography (HPLC) and gas chromatography (GC) coupled with mass spectrometry (LC-MS and GC–MS) have typically been used to analyze food components (Ares et al., 2018, Rocchetti et al., 2019). These methods can detect trace components owing to their high sensitivity. However, they require an authentic sample for each compound for accurate quantification, and some components can be altered or lost during pretreatment. Moreover, the functionality conferred by intermolecular interactions among the components cannot be properly evaluated.

In recent years, nuclear magnetic resonance (NMR) has also been applied in food analysis, which can discern the chemical properties and intermolecular interactions of complex mixtures (Schievan et al., 2019).

In this study, we used comprehensive metabolic profiling by NMR and flavonoid profiling by HPLC to analyze commercially available bee pollen products from different countries. By combining the advantages of these methods, we identified potential chemical markers to characterize the geographical origins of different pollen samples from their specific fingerprints. The information obtained in this study is expected to broaden the understanding of this food matrix and provide further indications of its health-promoting properties and should lead to the development of new quality control methods for bee pollen products.

## 2 Materials and methods

### 2.1 Bee pollen samples and materials

**Fig. 1:**
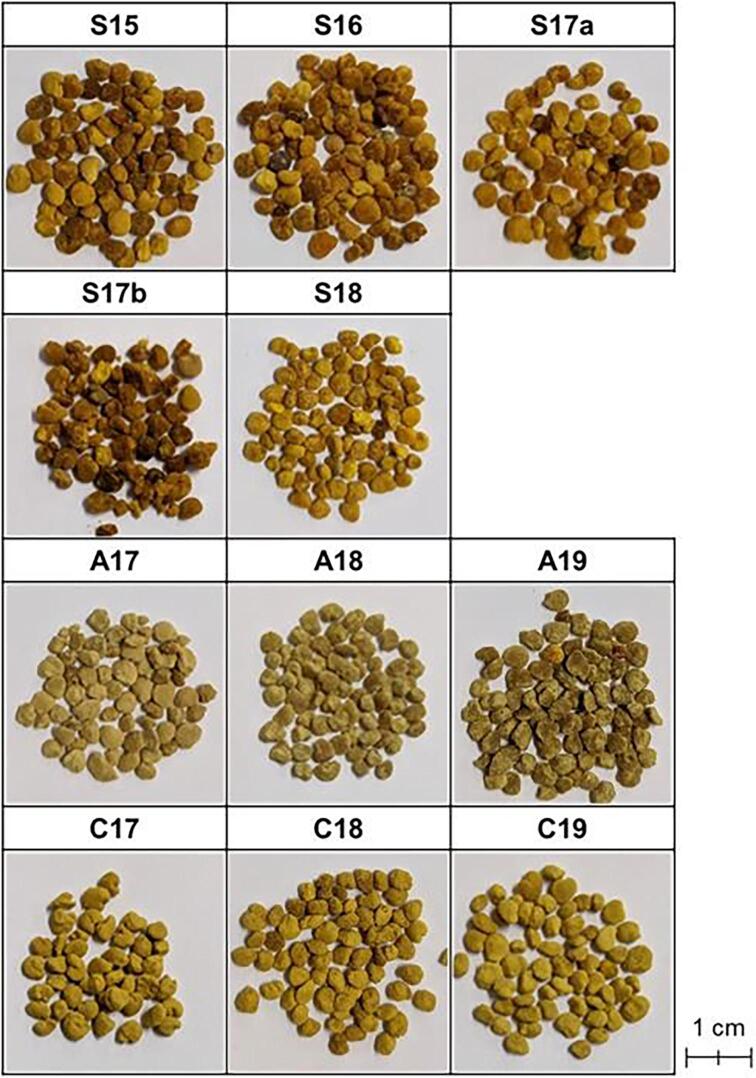
Appearances of the 11 kinds of bee pollen samples.

Bee pollen products sold by Api Co., Ltd. (Gifu, Japan) (https://www.api3838.co.jp/en) were used as the experimental samples. Samples from Spain were obtained from Reina Kilama, Sdad. Coop. Samples from Australia were obtained from Saxonbee Enterprises. Samples from China were obtained from Wuhan Honeycomb Healthy Co., Ltd. (Table 1 and Fig. 1).

**Table 1:** Detailed list of the bee pollen samples analyzed. The producing country, production year, identified botanical origins, and their ratios are provided.

Sample	Country	Year	Botanical Origin	Ratio (%)
S15	Spain	2015	Cistaceae, Cistaceae	39
			Echium, Boraginaceae, Bugloss	20
			Quercus ilex-T, Fagaceae, Evergreen Oak-T	8
			Rubus-T, Rosaceae, Bramble	4
S16	Spain	2016	Cistaceae, Cistaceae	53
			Echium, Boraginaceae, Bugloss	26
			Rubus-T, Rosaceae, Bramble	4
			Cruciferae, Cruciferae, Crucifers	3
S17a	Spain	2017	Cistaceae, Cistaceae	49
			Trifolium-T, Leguminosae, Clover-T	12
			Quercus, Fagaceae, Oak	4
			Amorpha, Leguminosae, False Indigo	3
S17b	Spain	2017	Cistaceae, Cistaceae	33
			Olea-T, Oleaceae, Olive	24
			Echium, Boraginaceae, Bugloss	10
			Rubus-T, Rosaceae, Bramble	4
			Quercus ilex-T, Fagaceae, Evergreen Oak-T	4
S18	Spain	2018	Echium, Boraginaceae, Bugloss	30
			Cistaceae, Cistaceae, Rock Rose	24
			Quercus, Fagaceae, Oak-nectarless	22
			Olea-T, Oleaceae, Olive	11
A17	Australia	2017	Eucalyptus-T, Myrtaceae, Gum-T	87
			Taraxacum-T, Compositae, Dandelion-T	9
A18	Australia	2018	Eucalyptus-T, Myrtaceae, Gum-T	90
			Taraxacum-T, Compositae, Dandelion-T	7
A19	Australia	2019	Eucalyptus-T, Myrtaceae, Gum-T	84
			Taraxacum-T, Compositae, Dandelion-T	8
			Myrtaceae, Myrtle Family	8
C17	China	2017	Cruciferae, Cruciferae, Crucifers	89
			Umbelliferae, Umbellifers	6
C18	China	2018	Cruciferae, Cruciferae, Crucifers	91
			Chenopodiaceae, Goosefoot family	4
C19	China	2019	Cruciferae, Cruciferae, Crucifers	86
			Pterocarya, Juglandaceae, Wingnut	10

The bee pollen samples had been prepared by the producers as follows. Pollen collected by bees was obtained from the hive and dried in a dryer (45 °C for Spain and 42 °C for Australia) or dried in open air using sunlight (China) to control the moisture content to under 8% (for Spain and China) or 5–6% (for Australia). Foreign substances such as bee parts and other hive debris were then removed. The samples were kept in shaded plastic bags in the dark at room temperature until use in the experiments. The botanical origin compositions of the bee pollen samples was determined using the Melissopalynology method (Louveaux et al., 1978) by an analytical institution (Intertek Food Services GmbH, Bremen, Germany). The production country, production year, and botanical origin composition of all samples are shown in Table 1, Table 2.

**Table 2:** Detailed information about origins and suppliers of bee pollen samples.

Country	Supplier	Producing year/area
Spain	Reina Kilama, Sdad. Coop.^1^	2015/Salamanca 2016/Toledo 2017/Caceres 2018/Salamanca
Australia	Saxonbee Enterprises^2^	2017/Jarrahdale 2018/Darkan, Boyup Brook and Dale 2019/Dwellingup
China	Wuhan honeycomb Healthy Co., Ltd.^3^	2017/Qinghai 2018/Qinghai 2019/Qinghai

^1^https://www.reinakilama.com/en/.

^2^https://saxonbee.com.au/.

^3^https://www.fengzhichao.net/.

All other chemicals used in this study were purchased from Fujifilm Wako Pure Chemical Co. Ltd. (Osaka, Japan).

### 2.2 Preparation of pollen extracts

Water and methanol extracts of bee pollen samples were prepared for NMR analysis as follows: For water extraction, each sample (200 mg) of finely ground bee pollen was vortexed (Vortex Genie-2, Scientific Industries, Bohemia, NY, USA) with 1 mL deuterium oxide (D_2_O) for 5 min at room temperature in a 1.5 mL polypropylene microcentrifuge tube (131-715CS-N, Watson, Tokyo, Japan) and incubated at 60 °C for 15 min. The mixture was vortexed for 5 min, incubated for 15 min, and vortexed for another 5 min. For methanol extraction, each sample (200 mg) of finely ground bee pollen was vortexed with 1 mL of 100% methanol‑*d_4_* (CD_3_OD) for 5 min at room temperature in a 1.5 mL microcentrifuge tube (131-715CS-N, Watson), incubated at 60 °C for 15 min, and vortexed again for 5 min at room temperature. After extraction, the samples were centrifuged for 15 min at 15,000 × g at 20 °C, and the unfiltered supernatants were used as the bee pollen D_2_O extract and bee pollen CD_3_OD extract. Each pollen sample was extracted in triplicate. 3-(trimethylsilyl)propionic acid sodium salt (TSP)-*d_4_* (200 mM, 10 µL) was added to 590 µL of each extract as an internal reference. Then, each sample (total 600 µL) was transferred into a 5 mm ϕ NMR tube made of DURAN borosilicate glass (PS-005, Shigemi, Tokyo, Japan) and stored at 4 °C until NMR measurement.

Methanol extracts of the bee pollen samples for HPLC analysis were prepared as follows: Each sample of finely ground pollen (200 mg) was vortexed with 1 mL 100% methanol (CH_3_OH, 99.8%, Fujifilm Wako Pure Chemical, Tokyo, Japan) for 5 min at room temperature, incubated at 60 °C for 15 min, and vortexed for 5 min at room temperature. After extraction, the samples were centrifuged for 15 min at 15,000 × g and 20 °C. The supernatant was filtered through a 0.45 μm PVDF membrane filter (Cosmonice filter 06543–04, Nacalai Tesque, Kyoto, Japan) to obtain the bee pollen CH_3_OH extract. Each pollen sample was extracted in triplicate. The extracts were shielded from light and stored at 4 °C until further analysis. HPLC analysis was conducted within 12 h of the extraction procedure.

### 2.3 NMR measurements

NMR measurements were performed at 20 °C using a Unity INOVA-500 spectrometer (Agilent Technologies, Santa Clara, CA, USA). Quantitative ^1^H NMR spectra were measured at 499.87 MHz to quantify the major components; the HDO signal was suppressed by pre-saturation. The TSP-*d_4_* signal was used as an internal reference, and its chemical shift was set to 0 ppm. For quantification, the acquisition parameters were set as follows: spectral width, 6,000 Hz; number of data points, 32 k; acquisition time, 2.731 s; delay time, 20 s; and number of scans, 32. The delay time (d1) was set to 20 s for all bee pollen samples dissolved in D_2_O or CD_3_OD based on the results of the inversion recovery method for the measurement of *T*_1_ (the longitudinal relaxation time), in which the interval time (d2) between the 180° pulse and 90° pulse was set to 0.0125, 0.025, 0.05, 0.1, 0.2, 0.4, 0.8, 1.6, 3.2, 6.4, 12.8, and 25.6 s, while the acquisition time (aq) was set to 2.73 s. The value of *T*_null_, *i.e.*, the d2 value at which the NMR signal is null, was less than 3.2 s for all the bee-pollen-derived ^1^H NMR signals in both D_2_O and CD_3_OD. Thus, d1 was set to 20 s based on the following equations:

d1 ≥ 5 × *T*_1_ – aq.

*T*_1_ = √2 × *T*_null_.

For NMR signal assignments, ^13^C NMR and two-dimensional NMR spectra (^1^H–^1^H DQF-COSY, ^1^H–^13^C HSQC, and ^1^H–^13^C HMBC) of the bee pollen sample extracts were measured.

^13^C NMR spectra were measured at 125.71 MHz. The acquisition parameters of the ^13^C NMR spectrum were as follows: spectral width, 47,348 Hz; number of data points, 64 K; acquisition time, 0.692 s; delay time, 2.0 s; and number of scans, 66,105.

For DQF-COSY, the acquisition time and delay time were set at 0.171 s and 2.0 s, respectively. The spectral widths were 6,000 Hz (F1) and 6,000 Hz (F2), and the number of data points and scans were set to 256 (F1), 2,048 (F2), and 64.

The ^1^H–^13^C HSQC spectra were generated in phase-sensitive mode with the following acquisition parameters: spectral width, 6,000 Hz for ^1^H and 24,510 Hz for ^13^C; number of data points, 2048 for ^1^H and 256 for ^13^C; acquisition time, 0.171 s; delay time, 2.0 s; and number of scans, 64.

The ^1^H–^13^C HMBC spectra were measured in absolute mode with the following parameters: spectral width, 6,000 Hz for ^1^H and 32,000 Hz for ^13^C; number of data points, 2048 for ^1^H and 256 for ^13^C; acquisition time, 0.171 s; delay time, 2.0 s; and number of scans, 128.

Preprocessing of the free induction decays (FIDs) and subsequent Fourier transformations were performed using the program MestRe Nova 12.0 (MestRec, Santiago de Compostela, Spain). Phase correction, baseline correction, and reference setting were performed manually. NMR signals were analyzed by comparing them with previously published NMR assignments and composition data and by referring to the online NMR database Biological Magnetic Resonance Data Bank (BMRB). The signals were then confirmed and assigned to the candidate compounds based on the ^1^H, ^13^C, and 2D NMR spectra and the results of spiking experiments. The TSP-*d_4_* signal (final concentration: 370 μM) was used as a reference to determine the concentration of each component by integrating the peak and calculating its ratio with the peak area of the reference.

### 2.4 HPLC profiling of flavonoids

The comprehensive flavonoid profiles of the bee pollen samples were obtained using HPLC with a Prominence HPLC system (Shimadzu, Kyoto, Japan). The HPLC conditions for the screening of flavonoid compounds were optimized according to previous research (Duan et al., 2019). Chromatographic separation was achieved using a PEGASIL ODS SP100 column (250 mm × 4.6 mm i.d., 5 μm; Senshu Scientific Co., Ltd., Tokyo, Japan) connected to a PEGASIL ODS SP100 guard column (30 mm × 4.6 mm i.d., 5 μm; Senshu Scientific Co., Ltd.). Solvent A was 99% water (Milli-Q), 1% acetonitrile, and 0.2% acetic acid, and solvent B was 99% acetonitrile, 1% water (Milli-Q), and 0.16% acetic acid. The following linear gradient was applied at a flow rate of 0.8 mL/min: 0–10.4 min (2 CV), 5% solvent B; 10.4–15.6 min (3 CV), 5–15% B; 15.6–41.5 min (5 CV), 15–17% B; and 41.5–103.8 min (12 CV), 17–41% B. The injection volume was 100 µL. The chromatograms were monitored at three wavelengths: 285, 330, and 360 nm. The absorbance values at the three wavelengths with a retention time range of 15–103 min were exported for further statistical and chemometric analyses.

### 2.5 Statistics and chemometrics

The concentrations of the quantified compounds were used to generate a heat map using the program MetaboAnalyst 4.0 (Chong et al., 2018). The quantitative ^1^H NMR spectrum of each bee pollen sample was processed using the program MestRe Nova 12.0 (MestRec), excluding the spectral ranges of 4.75–4.90 ppm for the bee pollen D_2_O extract and 4.80–4.95 ppm for the bee pollen CD_3_OD extract, respectively, which are the regions containing the water signal. The region 0.06–9.40 ppm was binned into strips with a width of 0.04 ppm. The raw data of the metabolic profiles of the different bee pollen samples were analyzed using the free software program R (version 3.6.0). Unsupervised principal component analysis (PCA), supervised orthogonal partial least squares discriminant analysis (OPLS-DA), and partial least squares discriminant analysis (PLS-DA) were then performed. In the multivariate analyses, all data were mean-centered and divided by the standard deviation as auto-scaling, but without normalization.

The dataset of the retention time (15–103 min) and absorbance of the chromatograms at the three wavelengths from HPLC was exported for statistical and chemometric analyses. The chromatogram data of the flavonoid profiles of the different bee pollen samples were processed using the software program R (version 3.6.0) after auto-scaling as described above, followed by unsupervised PCA.

Statistical significance was analyzed by one-way ANOVA followed by Tukey’s honestly significant difference (HSD) post-hoc test using the program R (Version 3.6.0). p ≤ 0.05 was considered to be statistically significant. All experiments were performed in triplicate, unless otherwise indicated.

## 3 Results

### 3.1 Quantitative Analysis of Components by NMR

**Fig. 2:**
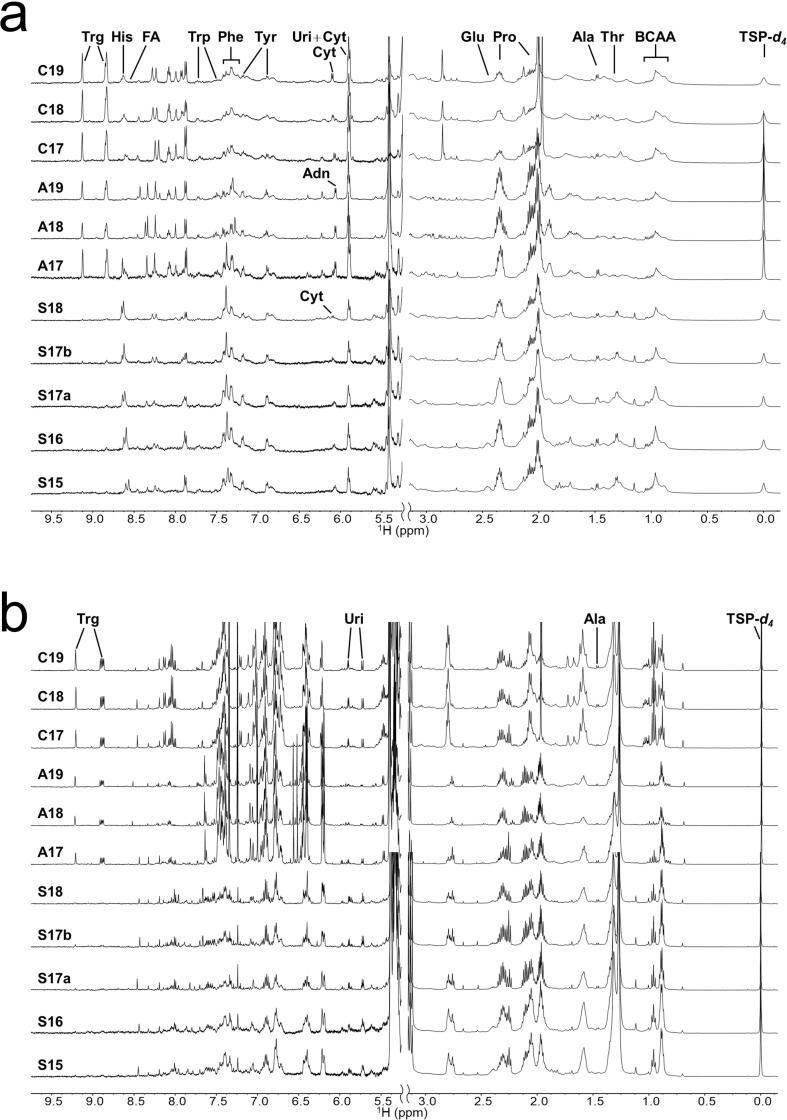
^1^H NMR spectra of the high (0.0–3.0 ppm) and low magnetic field (5.5–9.5 ppm) regions, and signal assignment of the components in bee pollen D_2_O extracts (a) and bee pollen CD_3_OD extracts (b).

Enlarged views of the high magnetic field region (0.8–3.0 ppm) and the low magnetic field region (5.5–9.5 ppm) of the ^1^H NMR spectra of the D_2_O and CD_3_OD extracts of the 11 types of bee pollen are shown in Fig. 2a and 2b, respectively. The complete ^1^H NMR spectra and ^13^C spectra for the S16 bee pollen D_2_O and CD_3_OD extracts are shown in Figs. S1 and S2, respectively. The ^1^H NMR spectra indicated that the bee pollen samples were rich in monosaccharides such as fructose and glucose. They also contained observable minor components, which were identified as amino acids, organic acids, nucleosides, and an alkaloid. The ^13^C spectra indicated that the S16 bee pollen sample contained substantial amounts of amino acids, mainly hydrophobic amino acids, in addition to monosaccharides. The NMR signals for the 24 components in the bee pollen D_2_O extracts were assigned by analyzing the 1D and 2D NMR spectra (Figs. S3–S5) and spiking experiments; they were identified as acetic acid, adenosine, alanine, arginine, cytidine, formic acid, fructose, α-glucose, β-glucose, glutamate, histidine, isoleucine, leucine, lysine, methionine, phenylalanine, proline, sucrose, threonine, trigonelline, tryptophan, tyrosine, uridine, and valine. The NMR signals for five components of the bee pollen CD_3_OD extracts were assigned to acetic acid, α-glucose, β-glucose, trigonelline, and uridine. The assignment list for the bee pollen D_2_O extracts is presented in Table S1.

**Fig. 3:**
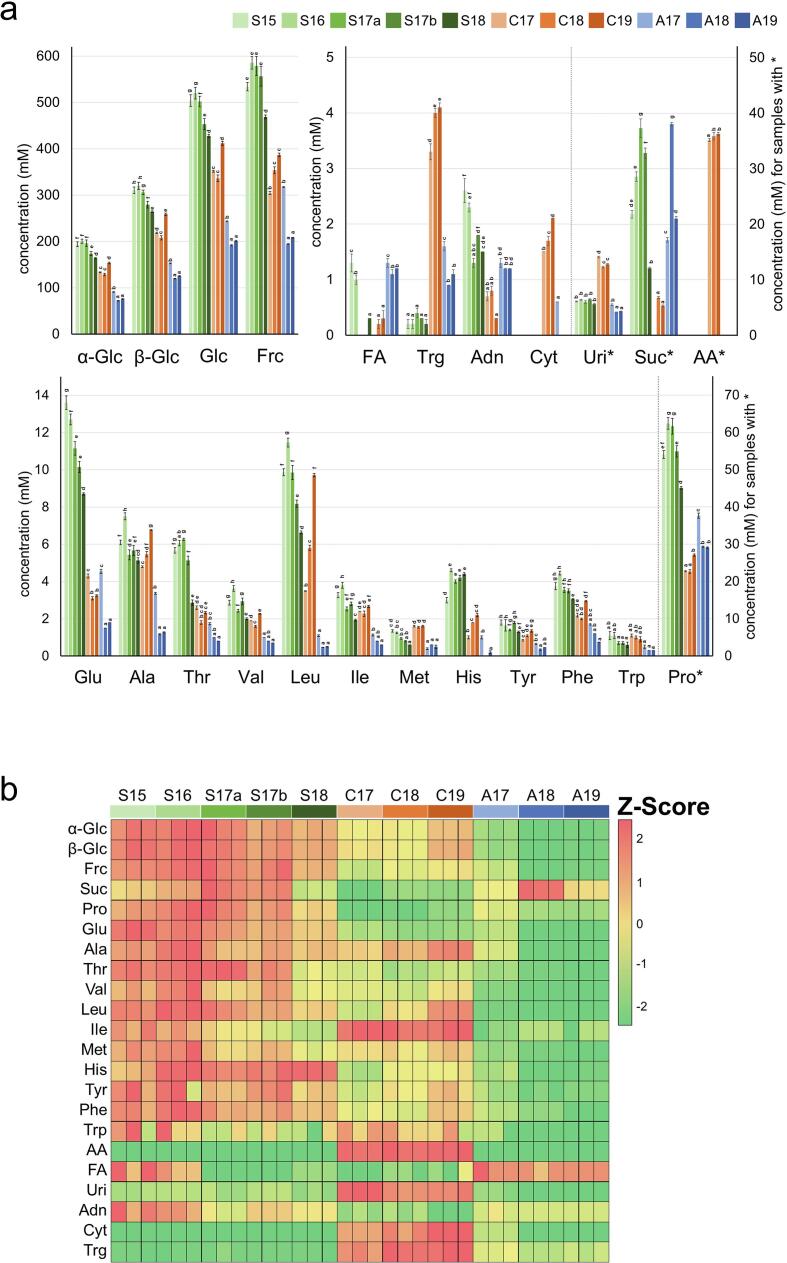
Concentrations of 22 components in the 11 types of bee pollen D_2_O extracts determined using quantitative ^1^H NMR spectroscopy (a), and the generated heat map (b). The data in a are presented as mean values ± SD (n = 3). The left and right vertical axes in a represent components without and with *, respectively. Means with the same letter are not significantly different from each other (*p* less than 0.05). The scale bar in b represents the Z-Score of each chemical compound in each sample.

The concentrations of 22 of the assigned components (excluding arginine and lysine, whose signals overlapped with other signals) in the 11 types of bee pollen D_2_O extracts were quantified by integrating the peak areas of the ^1^H NMR spectra. Three samples for NMR measurement were prepared in three NMR tubes for each type of bee pollen sample. Each sample was measured once, and the concentrations of the components were calculated as the mean value of the three measurement results. The calculated concentrations of these 22 components are shown in Fig. 3a. Additionally, the heat map generated from the quantitative data is shown in Fig. 3b.

Sugars, mainly D-glucose and D-fructose, are known to be the most abundant components among bee pollen nutrients (Campos et al., 2010, Komosinska-Vassev et al., 2015). The bee pollen extracts from Spain contained more D-glucose than those from the other two countries: Spain, 428–520 mM; China, 336–412 mM; and Australia, 192–243 mM (combined α-D-glucose and β-D-glucose). The concentration of β-D-glucose was 1.5 to 1.7 times higher than that of α-D-glucose in all the bee pollen extracts. In terms of D-fructose content, the bee pollen extracts from Spain contained the highest amount: 469–586 mM; China, 304–387 mM; and Australia, 194–318 mM. Sucrose was detected as a minor sugar component in all the bee pollen samples except C17. The bee pollen extracts from Spain and Australia contained more sucrose than those from China: Spain, 1.21–3.73 mM; China, 0–0.68 mM; and Australia, 1.72–3.80 mM.

Proline was the most abundant amino acid in all the bee pollen extracts, which is consistent with previous reports (Paramás et al., 2006, Serra Bonvehí and Escolà Jordà, 1997, Yang et al., 2013). The proline concentrations ranged from 22.7 to 62.4 mM. Trigonelline, an alkaloid, is a constituent of aroma and flavor in foods such as coffee, and it has been reported to have antimicrobial, anti-carcinogenic, anti-hyperglycemic, and anti-degranulation effects (Nugrahini et al., 2020). The trigonelline concentrations in the bee pollen extracts from China were the highest, whereas those in the bee pollen extracts from Spain were the lowest. A high concentration of acetic acid was detected only in the bee pollen extracts from China. Nucleosides, such as uridine, adenosine, and cytidine, showed characteristic results depending on the country of origin. Uridine, which has been reported to have anti-depressant, anti-epileptic, and brain-function-improving effects (Carlezon et al., 2005, Dobolyi et al., 2011), and cytidine were the most abundant in the bee pollen extracts from China, whereas adenosine was least abundant in the bee pollen extracts from China.

### 3.2 Multivariate analysis of ^1^H NMR data

**Fig. 4:**
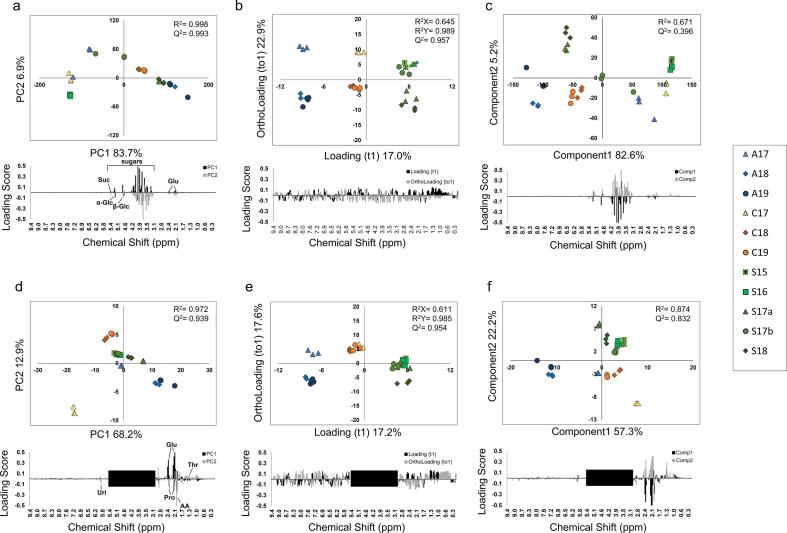
Principal component analysis of the metabolic profile of D_2_O extracts of bee pollen samples from three different countries determined using NMR. Score plot and loading plot for PCA using the entire ^1^H NMR spectrum (a). Score plot and loading plot for OPLS-DA using the entire ^1^H NMR spectrum (b). Score plot and loading plot for PLS-DA using the entire ^1^H NMR spectrum (c). Score plot and loading plot for PCA using the ^1^H NMR spectrum excluding the sugar region (3.0–5.5 ppm) (d). Score plot and loading plot for OPLS-DA using the ^1^H NMR spectrum excluding the sugar region (3.0–5.5 ppm) (e). Score plot and loading plot for PLS-DA using the ^1^H NMR spectrum excluding the sugar region (3.0–5.5 ppm) (f). R^2^ measures the goodness of fit in the PCA and PLS-DA models. R_x_^2^ and R_y_^2^ measure the goodness of fit in the OPLS-DA models. Q^2^ indicates the predictive ability of the models.

First, PCA (unsupervised), OPLS-DA (supervised) and PLS-DA (supervised) were applied to the dataset of the integrated ^1^H NMR spectra of 11 bee pollen D_2_O extracts from Spain, China, and Australia containing quantitative data. For the dataset containing the information of the entire ^1^H NMR spectra, the PCA (Fig. 4a), OPLS-DA (Fig. 4b), and PLS-DA (Fig. 4c) results are shown as two-dimensional score plots. In the PCA, PC1 explained 83.7% and PC2 explained 6.9% of the variance. The quality of the PCA model was described by R^2^ (0.998) and Q^2^ (0.993) values (Fig. 4a). Sugar contributed most to the grouping of the samples. However, the plots of bee pollen from the three countries overlapped and were not properly grouped. In the OPLS-DA analysis, the loading (t1) explained 17.0% and the ortholoading (to1) explained 22.9% of the variance. The quality of the OPLS-DA model was described by R_x_^2^ (0.645), R_y_^2^ (0.989), and Q^2^ (0.957) values (Fig. 4b). All samples were properly grouped, but the loading scores showed that the significant molecules for metabolic signatures were unclear. In the PLS-DA analysis, Component1 explained 82.6% and Component2 explained 5.2% of the variance. The quality of the PLS-DA model was described by R^2^ (0.671) and Q^2^ (0.396) values (Fig. 4c). The results were similar to those of the PCA analysis. However, the prediction quality was relatively low. Sugar contributed the most to the grouping of the samples.

The sugar region (3.0–5.5 ppm) was then excluded from the dataset, and the resulting PCA, OPLS-DA, and PLS-DA score plots are shown in Fig. 4d, e, and f. In the PCA, PC1 explained 68.2% and PC2 explained 12.9% of the variance. The quality of the PCA model was described by R^2^ (0.972) and Q^2^ (0.939) values. However, samples A17, which were from Australia, were not properly grouped, instead overlapping with samples from Spain. From the loading plot (Fig. 4d), acetic acid, proline, and glutamate were found to contribute to the variance. In the OPLS-DA analysis, loading (t1) explained 17.2% and ortholoading (to1) explained 17.6% of the variance. The quality of the OPLS-DA model was described by R_x_^2^ (0.661), R_y_^2^ (0.985), and Q^2^ (0.954) values. All the samples were properly grouped, but the loading scores showed that the significant molecules for the metabolic signature were unclear (Fig. 4e). In the PLS-DA analysis, Component1 explained 57.3% and Component2 explained 22.2% of the variance. The quality of the PLS-DA model was described by R^2^ (0.874) and Q^2^ (0.832) values (Fig. 4f). The results of PLS-DA were similar to those of PCA.

**Fig. 5:**
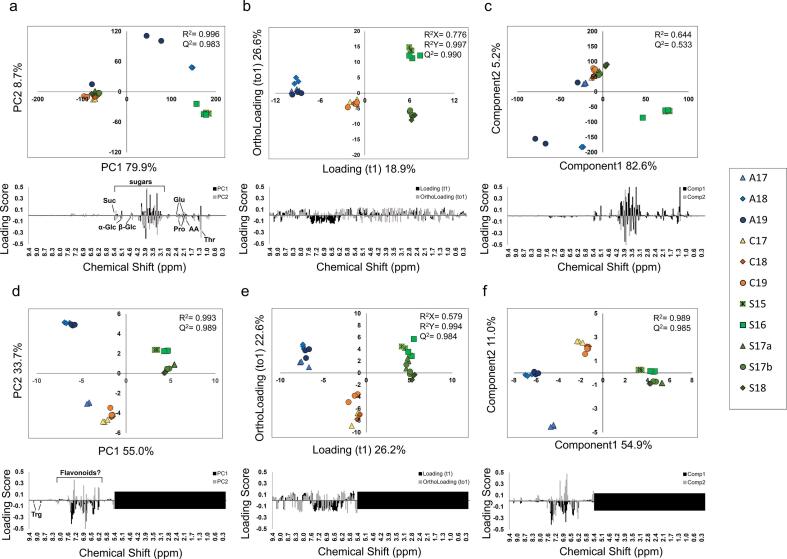
Principal component analysis of the metabolic profile of CD_3_OD extracts of bee pollen samples from three different countries determined using NMR. Score plot and loading plot for PCA using information from the entire ^1^H NMR spectrum (a). Score plot and loading plot for OPLS-DA using information from the entire ^1^H NMR spectrum (b). Score plot and loading plot for PLS-DA using information from the entire ^1^H NMR spectrum (c). Score plot and loading plot for PCA using the low magnetic field region (5.5–9.5 ppm) of the ^1^H NMR spectrum (d). Score plot and loading plot for OPLS-DA using the low magnetic field region (5.5–9.5 ppm) of the ^1^H NMR spectrum (e). Score plot and loading plot for PLS-DA using the low magnetic field region (5.5–9.5 ppm) of the ^1^H NMR spectrum (f). R^2^ measures the goodness of fit in the PCA and PLS-DA models. R_x_^2^ and R_y_^2^ measure the goodness of fit in the OPLS-DA models. Q^2^ indicates the predictive ability of the models.

Next, the same analyses were applied to the dataset of the integrated ^1^H NMR spectra of the 11 types of bee pollen CD_3_OD extracts from the three countries (Fig. 5 abc). In the PCA, PC1 explained 79.9% and PC2 explained 8.7% of the variance. The quality of the PCA model was described by R^2^ (0.996) and Q^2^ (0.983) values. However, grouping based on the country of origin was not successful due to the overlap of the plots in the left panel. In the loading plot (Fig. 5a), the information in the sugar region seems to provide the main contribution, as in the case of the D_2_O extracts. However, in the case of the bee pollen CD_3_OD extracts, proper grouping was still unsuccessful even after removing the information in the sugar region. In the OPLS-DA analysis, loading (t1) explained 18.9% and ortholoading (to1) explained 26.6% of the variance. The quality of the OPLS-DA model was described by R_x_^2^ (0.776), R_y_^2^ (0.997), and Q^2^ (0.990) values. All the samples were properly grouped, but the loading score showed that the significant molecules for the metabolic signature were unclear (Fig. 5b). In the PLS-DA analysis, Component1 explained 82.6% and Component2 explained 5.2% of the variance. The quality of the PLS-DA model was described by R^2^ (0.644) and Q^2^ (0.533) values (Fig. 5c). The results of PLS-DA were similar to those of PCA. However, the prediction quality of the PLS-DA model was lower than that of the PCA model.

Subsequently, the dataset containing the information from the polyphenol region (5.5–9.5 ppm), in which aromatic rings are observed, was used for further analysis. The resulting two-dimensional score plots are shown in Fig. 5d, e, and f. In the PCA, PC1 explained 55.0% and PC2 explained 33.7% of the variance. The quality of the PCA model was described by R^2^ (0.993) and Q^2^ (0.989) values (Fig. 5d). The samples were divided into three groups according to the countries of origin of the bee pollen samples. Notably, the A17 samples were located closer to the Chinese samples than the other Australian samples. In the OPLS-DA analysis, loading (t1) explained 26.2% and ortholoading (to1) explained 22.6% of the variance. The quality of the OPLS-DA model was described by R_x_^2^ (0.579), R_y_^2^ (0.994), and Q^2^ (0.984) values. All the samples were properly grouped, but the loading score showed that the significant molecules for the metabolic signature were unclear (Fig. 5e). In the PLS-DA analysis, Component1 explained 54.9% and Component2 explained 11.0% of the variance. The quality of the PLS-DA model was described by R^2^ (0.989) and Q^2^ (0.085) values (Fig. 5c). The results of PLS-DA were similar to those of PCA. However, the prediction quality of the PLS-DA model was lower than that of the PCA model.

### 3.3 Multivariate analysis of HPLC data

**Fig. 6:**
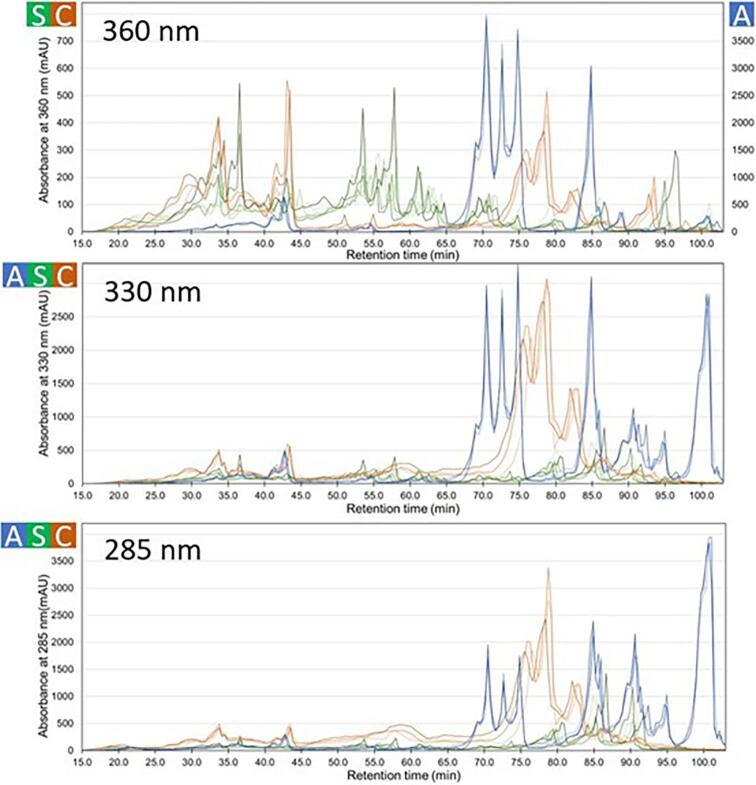
Overview of the chromatograms obtained by HPLC analysis for the 11 types of bee pollen. CH_3_OH extracts detected at 360 nm, 330 nm, and 285 nm. S, C and A on the vertical axis indicate that the axis corresponds to bee pollen samples from Spain, China, and Australia, respectively. In the top figure, the left vertical axis is for the eight bee pollen samples from Spain and China, and the right vertical axis is for the three bee pollen samples from Australia.

The flavonoid components in the CH_3_OH extracts of the bee pollen samples were analyzed using HPLC, in which the absorbances at 360, 330, and 285 nm were monitored to detect mainly flavonols, flavones, and flavanones, respectively. The chromatograms obtained for each wavelength are shown in Fig. 6. The bee pollen samples from the three countries showed characteristic chromatograms at each of the three wavelengths, whereas the bee pollen samples from different years from the same country showed similar chromatograms (Fig. 6). The bee pollen extracts from Spain showed a greater number of peaks and more varied patterns among the bee pollen samples collected in different years than the bee pollen extracts from China and Australia. The chromatogram at 360 nm showed particularly complex patterns in the bee pollen extracts from Spain. However, the chromatogram patterns of the bee pollen extracts from China and Australia were very similar, even though they were produced in different years, and there was almost no variation among the three experiments.

**Fig. 7:**
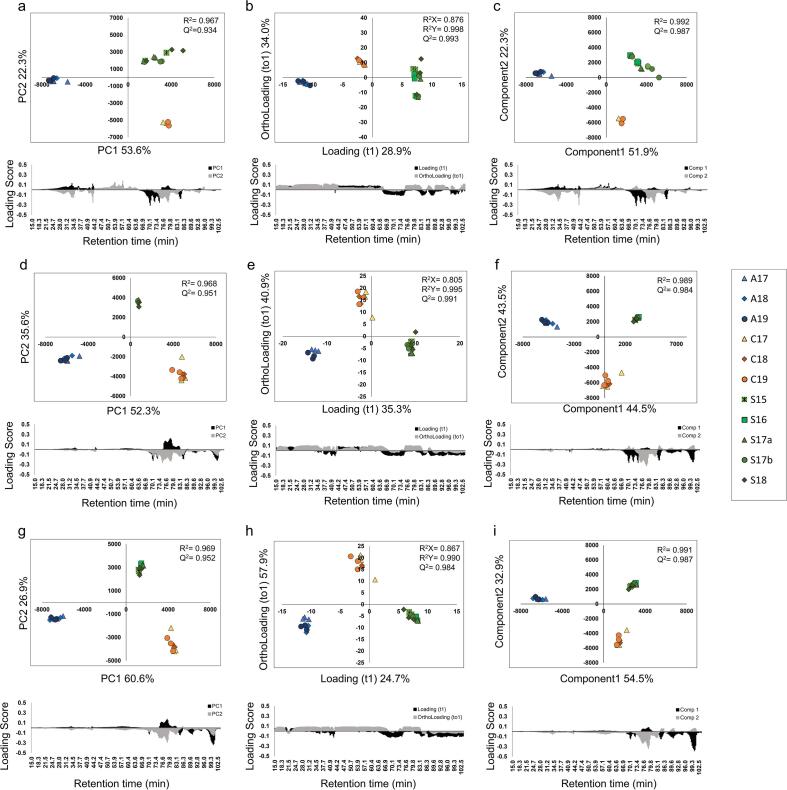
Principal component analysis of the flavonoid profile obtained by HPLC for the CH_3_OH extracts of bee pollen from three different countries. a: Score plot and loading plot for PCA using the absorbance at 360 nm. b: Score plot and loading plot for OPLS-DA using the absorbance at 360 nm. c: Score plot and loading plot for PLS-DA using the absorbance at 360 nm. d: Score plot and loading plot for PCA using the absorbance at 330 nm. e: Score plot and loading plot for OPLS-DA using the absorbance at 330 nm. f: Score plot and loading plot for PLS-DA using the absorbance at 330 nm. g: Score plot and loading plot for PCA using the absorbance at 285 nm. h: Score plot and loading plot for OPLS-DA using the absorbance at 285 nm. i: Score plot and loading plot for PLS-DA using the absorbance at 285 nm. R^2^ measures the goodness of fit in the PCA and PLS-DA models. R_x_^2^ and R_y_^2^ measure the goodness of fit in the OPLS-DA models. Q^2^ indicates the predictive ability of the models.

The results of applying PCA and OPLS-DA to the chromatogram dataset obtained at each wavelength are shown in Fig. 7. In the PCA analysis, for the 360 nm dataset, PC1 explained 53.6%, and PC2 explained 22.3% of the variance (Fig. 7a). The quality of the PCA model was described by R^2^ (0.967) and Q^2^ (0.934) values. For the 330 nm dataset, PC1 explained 52.3%, and PC2 explained 35.6% of the variance (Fig. 7d). The quality of the PCA model was described by R^2^ (0.968) and Q^2^ (0.951) values. For the 285 nm dataset, PC1 explained 60.6% and PC2 explained 26.9% of the variance (Fig. 7g). The quality of the PCA model was described by R^2^ (0.969) and Q^2^ (0.952) values. The bee pollen extracts from each country were convergently placed in all score plots for each wavelength. Thus, appropriate grouping according to country of origin was successful.

In the OPLS-DA analysis, for the 360 nm dataset, loading (t1) explained 28.9% and ortholoading (to1) explained 34.0% of the variance (Fig. 7b). The quality of the PCA model was described by R_x_^2^ (0.876), R_y_^2^ (0.998), and Q^2^ (0.993) values. For the 330 nm dataset, loading (t1) explained 35.3% and ortholoading (to1) explained 40.9% of the variance (Fig. 7e). The quality of the PCA model was described by R_x_^2^ (0.805), R_y_^2^ (0.995), and Q^2^ (0.991) values. For the 285 nm dataset, loading (t1) explained 24.7% and ortholoading (to1) explained 57.9% of the variance (Fig. 7h). The quality of the PCA model was described by R_x_^2^ (0.867), R_y_^2^ (0.990), and Q^2^ (0.984) values. All samples in each dataset were properly grouped, but the loading scores showed that the significant patterns for metabolic signatures were unclear.

In the PLS-DA analysis, for the 360 nm dataset, Component1 explained 51.9% and Component2 explained 22.3% of the variance (Fig. 7c). The quality of the PLS-DA model was described by R^2^ (0.992) and Q^2^ (0.987) values. For the 330 nm dataset, Component1 explained 44.5% and Component2 explained 43.5% of the variance (Fig. 7f). The quality of the PLS-DA model was described by R^2^ (0.989) and Q^2^ (0.984) values. For the 285 nm dataset, Component1 explained 54.5% and Component2 explained 32.9% of the variance (Fig. 7i). The quality of the PLS-DA model was described by R^2^ (0.991) and Q^2^ (0.987) values. The results of PLS-DA were similar to those of PCA.

## 4 Discussion

Bee pollen is rich in nutrients and is expected to be a nutritionally functional food for humans; however, its composition varies depending on the plant source (Denisow and Denisow-Pietrzyk, 2016, Feas et al., 2012). In this study, commercially available bee pollen products from multiple lots were collected over several years from three different countries, and their geographical origin compositions were identified and analyzed.

Recently, many studies have been conducted on the functionality and component analysis of bee pollen using HPLC as an effective analytical method (Ares et al., 2018). However, there is increasing interest in the use of NMR, the application of which has experienced exponential growth in several research fields, particularly food science, for geographical origin characterization, aging analysis, and fraud detection (Consonni & Cagliani, 2019). For example, NMR has been used to profile other beehive products such as honey and propolis (Maraschin et al., 2016, Schievano et al., 2016, Wang et al., 2020). To the best of our knowledge, although solid-state NMR analysis of bee pollen has been reported (Paradowska et al., 2017), there have been no studies on the component profiling of liquid bee pollen extracts by NMR.

In this study, the samples from China and Australia originated from relatively similar plants (Table 1). However, for the samples from Spain, the composition of the originating plants varies from year to year. Thus, chemical composition analysis is necessary to reveal differences at the molecular level. Evaluation of the bee pollen extracts using NMR and HPLC revealed that their composition differed greatly depending on their country of origin.

Interestingly, multivariate analyses demonstrated that classification by country of origin was possible, even when the year of production was different. In our multivariate analyses, unsupervised PCA, supervised OPLS-DA, and PLS-DA were performed.

Among the three methods, OPLS-DA provided the best grouping model, but based on the loading score, we were unable to identify the signature components in each bee pollen sample (Fig. 4, Fig. 5, Fig. 7). Therefore, OPLS-DA was inappropriate for the analysis of bee pollen samples.

PLS-DA provided a similar model to PCA, and the PLS-DA models for the HPLC results were relatively good. However, in the case of the NMR results, the models provided by PLS-DA showed low credibility. For example, the prediction quality of the PLS-DA model is occasionally low (Fig. 4c and Fig. 5c), and bias may occur because PLS-DA is a supervised analysis (Fig. 5f). As a result, PLS-DA is less appropriate for the analysis of bee pollen samples than PCA. To reveal the original information of the bee pollen samples and avoid bias, we believe that unsupervised PCA is the appropriate model.

Although the PCA models show high goodness-of-fit (R^2^) and predictive power (Q^2^) values, the combined PC1 and PC2 scores in the PCA analyses vary from 75.9% to 90.6%, indicating limited representativeness due to the limited number of bee pollen samples used in this study. More samples from the three countries and production years are needed to characterize the bee pollen samples in detail.

However, it is still possible to characterize the bee pollen samples to some extent using the current PCA models. For example, grouping by year of production was not successful, but we managed to group by country of production using the NMR data (Fig. 4d and Fig. 5d).

In particular, using the data on flavonoids from HPLC, PCA was successful in grouping the bee pollen samples from Spain, in which the variability of the botanical origin was greater than those of the samples from China and Australia (Fig. 5d and 7adg). The source plants of the Spanish bee pollen samples varied yearly, but a certain pattern was observed: *Cistaceae* and *Echium* account for almost half of the samples (Table 1). The similarity in the composition and appearance of the Spanish bee pollen samples (Fig. 1) indicates that they have a similar chemical composition. The PCA results showed that the concentration of flavonoids was relatively constant in the Spanish bee pollen samples regardless of the year of production.

NMR quantitative analysis of the bee pollen D_2_O extracts showed that sugars, especially fructose and glucose, were the major components in all the bee pollen samples. The ratio of fructose to glucose (F/G value), which correlates with the glycemic index (GI) in honey, ranged from 1.01 to 1.30 in the bee pollen extracts from Spain and Australia and one bee pollen extract from China (C18), whereas the F/G values of the two other bee pollen extracts from China were 0.87 (C17) and 0.94 (C19), indicating that the amount of glucose exceeded the amount of fructose in these samples. In addition, high concentrations of trigonelline, nucleosides such as uridine and cytidine, and acetic acid were detected in the bee pollen extracts from China (Fig. 3b), and the information on these minor components, as well as on other amino acids and sugars, can be used to characterize bee pollen samples according to country of origin.

In the NMR analysis of the bee pollen CD_3_OD extracts, polyphenol signals were obtained (Fig. 2b), which could be used to characterize their country of origin. The potential grouping of the bee pollen samples based on the aromatic ring signals (Fig. 5d) suggests that the polyphenol compositions were mostly dissimilar among the bee pollen samples from different countries. However, NMR is not suitable for the quantitative analysis of the various flavonoids in bee pollen extracts, because the NMR signals of the flavonoids overlap significantly (Fig. 2).

However, HPLC can be used to separate and detect various the phenolic components with high sensitivity. Three wavelengths were used to detect the different groups of flavonoids: 360, 330, and 285 nm for flavonols, flavones, and flavanones, respectively. Using the high resolution and sensitivity of HPLC, the flavonoid profiles in the CH_3_OH extracts of the bee pollen samples were obtained (Fig. 6). In bee pollen samples from Australia, large peaks were observed at all three wavelengths, indicating that these samples were rich in all three groups of flavonoids, *i.e.*, flavonols, flavones, and flavanones. In particular, at 360 nm, only the bee pollen samples from Australia showed large peaks, indicating that flavonols were abundant in these samples. This could be attributed to the presence of eucalyptus, which is rich in flavonols. In contrast, in the bee pollen samples from China, large peaks were observed at 330 and 285 nm, indicating that these samples were rich in flavones and flavanones. In the case of the bee pollen samples from Spain, the peaks at the three wavelengths were relatively lower than those of the bee pollen samples from Australia and China, suggesting that the flavonoid content was lowest in the bee pollen samples from Spain.

The chromatographic data were then subjected to multivariate analysis to characterize the samples produced in different countries (Fig. 7adg). Based on the 360 nm datasets, the bee pollen samples from China and Australia converged and were successfully grouped, whereas those from Spain varied depending on the production year (Fig. 7a). This reflects the variation in the source plants of the Spanish bee pollen samples from different years. On the other hand, using the 285 and 330 nm datasets, all the bee pollen samples were successfully grouped according to country of origin. Thus, the composition of flavonols was found to vary due to the annual variation in the source plants in the Spanish bee pollen samples. However, the composition of flavones and flavanones in these samples was relatively constant.

The flavonoid profiles obtained by HPLC (Fig. 6) and the PCA results (Fig. 7adg) showed that the flavonoid compositions of the bee pollen samples varied among the samples from the three countries of origin, but were similar among the samples produced in different years in a certain country.

Although the bee pollen samples from Spain were extracted and analyzed as a whole without separation based on plant sources, the samples could still be characterized as being from Spain based on the NMR and HPLC analyses, indicating that the qualities of bee pollen remained relatively constant for each country of origin, even for different production years or lots. Further study of the use of NMR spectra of bee pollen CD_3_OD extracts and identification of the peaks of HPLC chromatograms may lead to a more detailed characteristic pattern for each country of origin.

## 5 Conclusion

In this analysis, both HPLC data, which mainly reflect information on flavonoids and polyphenolic compounds, and NMR data, which reflect data on amino acids, sugars, alkaloids, and nucleic acids, successfully predicted significant features of bee pollen samples from different countries. From a vendor’s perspective, the results obtained in this study could become the basis for quality stability between lots and lead to better quality control or guarantees when selling bee pollen products. From a consumer perspective, this study demonstrated that the quality of bee pollen products from the same country is relatively stable, whereas the components and functions of bee pollen products vary depending on the country of origin.

Furthermore, bee pollen products distributed on a commercial scale were analyzed, multiple lots of bee pollen from various countries and different years were compared, and a wide range of components were examined using both NMR and HPLC. The results are highly applicable for commercial use in the quality control of bee pollen.

## Declaration of Competing Interest

The authors declare that they have no known competing financial interests or personal relationships that could have appeared to influence the work reported in this paper.
