# Supplementary material for: NMR and HPLC profiling of bee pollen products from different countries
Source: Food Chem (Oxf). 2022 Jul 6;5:100119. doi: 10.1016/j.fochms.2022.100119 (PMC9278072; doi:10.1016/j.fochms.2022.100119)
Supplement: Supplementary data 1 [file mmc1.docx]

Supplementary data for:

**NMR and HPLC Profiling of Bee Pollen Products from Different Countries**

Peng Lu^a^, Saki Takiguchi^a^, Yuka Honda^a^, Yi Lu^a^, Taichi Mitsui^b^, Shingo Kato^b^, Rina Kodera^b^, Kazuo Furihata^c^, Mimin Zhang^a^, Ken Okamoto^a^, Hideaki Itoh^a^, Michio Suzuki^a^, Hiroyuki Kono^b^, Koji Nagata^ad*^

^a^ *Department of Applied Biological Chemistry,* ^c^ *Advanced Instrumental Analysis Unit and* ^d^ *Agricultural Bioinformatics Research Unit, Graduate School of Agricultural and Life Science, The University of Tokyo, 1-1-1 Yayoi, Bunkyo-ku, Tokyo 113-8657, Japan*

^b^ *Nagaragawa Research Center, API Co.,Ltd., 692-3 Nagara, Gifu-City, Gifu 502-0071, Japan*

* Corresponding author.

*E-mail address*: aknagata@mail.ecc.u-tokyo.ac.jp (K. Nagata).

Tel: +81 3 5841 1117; fax: +81 3 5841 5168

**Table S1** Assignments of ^1^H and ^13^C NMR signals of 21 components in D_2_O extract of bee pollens.

**Figure S1** The overall view of the ^1^H NMR spectrum of D_2_O extracts (a) and CD_3_OD extracts (b) of bee pollens.

**Figure S2** ^13^C NMR spectrum of S16 D_2_O extract of bee pollens. (a) the whole region, (b) the aliphatic region, and (c) the aromatic region.

**Figure S3** 2D NMR spectra of S16 D_2_O extract of bee pollens. (a-b) ^1^H-^1^H DQF-COSY (c-e) ^1^H-^13^C HSQC, and (f) ^1^H-^13^C HMBC spectra. (b, d, e) Zoomed spectra for the boxed regions in (a, c). Components characteristic to Spanish bee pollen samples are labeled in red.

**Figure S4** 2D NMR spectra of A18 D_2_O extract of bee pollens. (a-b) ^1^H-^1^H DQF-COSY (c-e) ^1^H-^13^C HSQC, and (f) ^1^H-^13^C HMBC spectra. (b, d, e) Zoomed spectra for the boxed regions in (a, c). Components characteristic to Australian bee pollen samples are labeled in red.

**Figure S5** 2D NMR spectra of C18 D_2_O extract of bee pollens. (a-b) ^1^H-^1^H DQF-COSY (c-e) ^1^H-^13^C HSQC, and (f) ^1^H-^13^C HMBC spectra. (b, d, e) Zoomed spectra for the boxed regions in (a, c). Components characteristic to Chinese bee pollen samples are labeled in red.

**Table S1** Assignments of ^1^H and ^13^C NMR signals of 21 components in D_2_O extract of bee pollens. The proton signals that were chosen for the quantitative analysis are indicated by *.

| Compound | Assignment | ^1^H (ppm) | Multiplicity | ^13^C (ppm) |
| --- | --- | --- | --- | --- |
| acetic acid | CH_3_* | 2.01 | s | 24.8 |
| adenosine | 2’CH(OH) | 4.78 | m | 76.5 |
|  | 1’CH* | 6.06 | d | 91.3 |
|  | 2CH | 8.25 | s | 155.4 |
|  | 8CH | 8.34 | s | 143.4 |
|  | 5C |  |  | 122.0 |
|  | 4C |  |  | 151.3 |
|  | 6C |  |  | 158.4 |
| alanine | β-CH_3_* | 1.48 | d | 18.9 |
| arginine | γ-CH_2_ | 1.67, 1.67 | m | 26.8 |
|  | β-CH_2_ | 1.91, 1.91 | m | 30.4 |
| cytidine | 1’CH | 5.91 | d | 92.0 |
|  | 5CH | 6.08 | d | 98.6 |
|  | 6CH | 7.89 | d | 144.5 |
| formic acid | H*COOH | 8.45 | s |  |
| fructose | 4CH* | 4.10 |  |  |
| α-glucose | 1CH* | 5.23 | d | 94.8 |
|  | 2CH | 3.54 | d | 74.2 |
|  | 3CH | 3.71 | d | 75.4 |
|  | 4CH | 3.41 | d | 72.3 |
|  | 5CH | 3.80 | t | 74.1 |
|  | CH_2_ |  | d | 63.3 |
| β-glucose | 1CH* | 4.64 | d | 98.6 |
|  | 2CH | 3.24 | d | 76.8 |
|  | 3CH | 3.48 | d | 78.8 |
|  | 4CH | 3.41 | d | 72.4 |
|  | 5CH | 3.48 | t | 78.6 |
|  | CH_2_ |  | d | 63.4 |

(To be continued)

Table S1 (Continued)

| Compound | Assignment | ^1^H (ppm) | Multiplicity | ^13^C (ppm) |
| --- | --- | --- | --- | --- |
| glutamine | β-CH_2_ | 2.14, 2.14 | m | 29.1 |
|  | γ-CH_2_* | 2.45, 2.45 | m | 33.5 |
| histidine | 4CH ring | 7.37 | s | 120.4 |
|  | 2CH* ring | 8.59 | s | 136.7 |
| isoleucine | δ-CH_3_* | 0.94 | t | 13.8 |
|  | γ-CH_3_ | 1.01 | d | 17.4 |
|  | γ-CH_2_ | 1.28, 1.43 | m | 27.2 |
|  | β-CH | 1.94 | m | 38.6 |
| leucine | δ-CH_3_* | 0.96 | d | 23.6 |
|  | δ’-CH_3_ | 0.96 | d | 24.7 |
|  | γ-CH | 1.72 | m | 26.9 |
|  | β-CH_2_ | 1.72, 1.72 | dd | 42.5 |
| lysine | γ-CH_2_ | 1.46, 1.46 | m | 24.1 |
|  | δ-CH_2_ | 1.70, 1.70 | dt | 29.0 |
|  | β-CH_2_ | 1.90, 1.90 | m | 32.6 |
|  | ε-CH_2_ | 3.02, 3.02 | t | 41.7 |
| methionine | CH_3_ | 2.13 | s | 16.6 |
|  | β-CH_2_ | 2.05, 2.19 | m | 32.3 |
|  | γ-CH_2_* | 2.63, 2.63 | t | 31.7 |
| phenylalanine | 2,6CH* ring | 7.32 | d | 132.1 |
|  | 4CH* ring | 7.38 | m | 130.4 |
|  | 3,5CH* ring | 7.41 | m | 131.8 |
|  | β-CH_2_ | 3.05, 3.21 | m | 39.0 |
| proline | γ-CH_2_ | 2.00, 2.00 | m | 26.5 |
|  | β-CH_2_ | 2.06, 2.35* | m | 31.7 |
|  | δ-CH_2_ | 3.33, 3.41 | m | 48.8 |
| sucrose | C*1H(Glc) | 5.41 | d | 94.8 |
|  | C4H(Frc) | 4.22 | d | 79.1 |
|  | CH_2_(Frc) | 4.05, 4.05 | dd | 76.8 |
| threonine | γ-CH_3_* | 1.33 | d | 22.1 |
|  | β-CH | 4.26 | q | 68.6 |

(To be continued)

Table S1 (Continued)

| Compound | Assignment | ^1^H (ppm) | Multiplicity | ^13^C (ppm) |
| --- | --- | --- | --- | --- |
| trigonelline | CH_3_ | 4.44 | s | 51.1 |
|  | 5CH | 8.08 | m | 130.5 |
|  | 6CH | 8.84 | d | 147.6 |
|  | 4CH | 8.84 | d | 148.9 |
|  | 2CH* | 9.13 | s | 148.7 |
| trigonelline | CH_3_ | 4.44 | s | 51.1 |
|  | CH-4 | 8.08 | m | 130.5 |
|  | CH-3 | 8.84 | d | 147.6 |
|  | CH-5 | 8.84 | d | 148.9 |
|  | CH*-1 | 9.13 | s | 148.7 |
| tryptophan | 6CH ring | 7.19 | m |  |
|  | 7CH ring | 7.28 | m |  |
|  | 2CH | 7.32 | s |  |
|  | 5CH ring | 7.51 | d |  |
|  | 4CH* ring | 7.72 | d |  |
| tyrosine | β-CH_2_ | 3.05, 3.21 | m | 38.2 |
|  | 2,6CH ring | 7.19 | d | 133.5 |
|  | 3,5CH* ring | 6.89 | d | 118.5 |
| uridine | 5CH* | 5.90 | d | 104.9 |
|  | 6CH | 7.88 | d | 144.5 |
|  | 1’CH | 5.90 | d | 92.0 |
|  | 2’CH(OH) | 4.40 | m | 76.1 |
| valine | γ’-CH_3_ | 0.99 | d | 19.4 |
|  | γ-CH_3_* | 1.04 | d | 20.7 |
|  | β-CH | 2.30 | m | 31.8 |

**(a)**


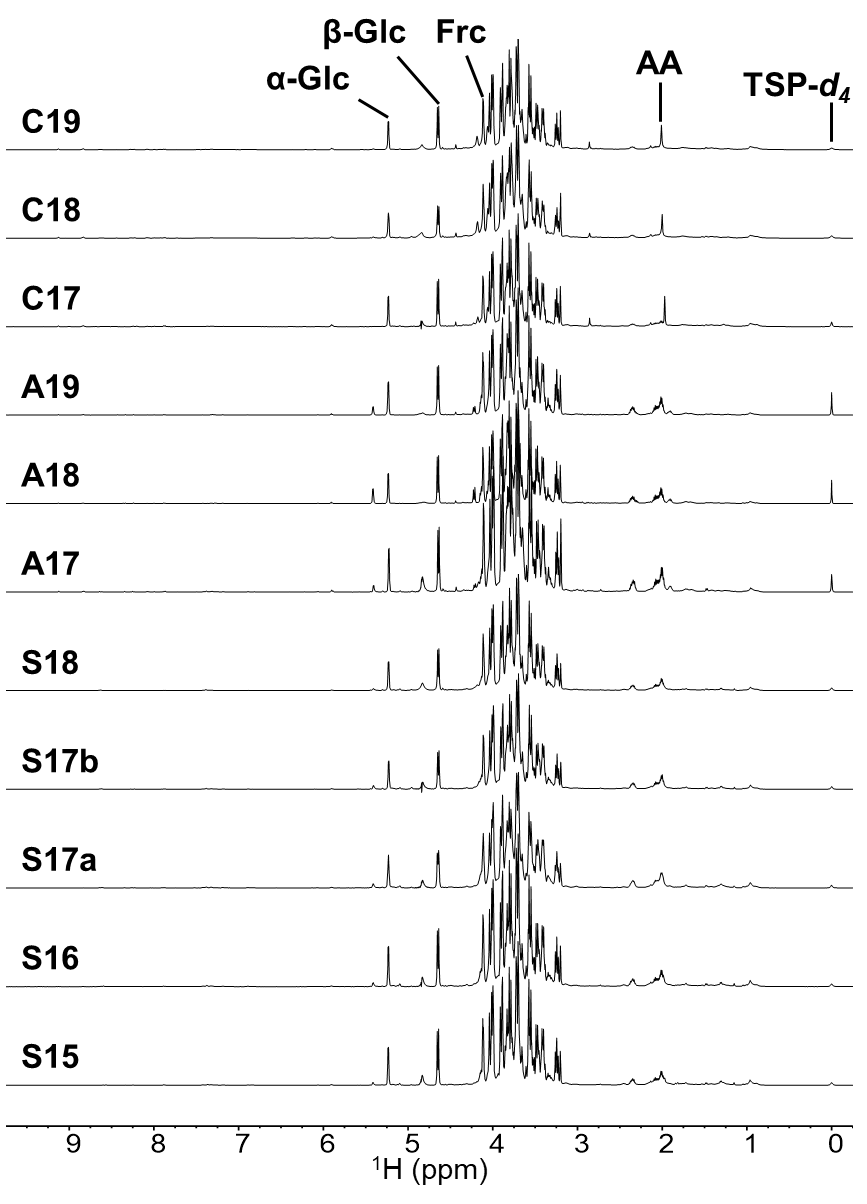


**(b)**


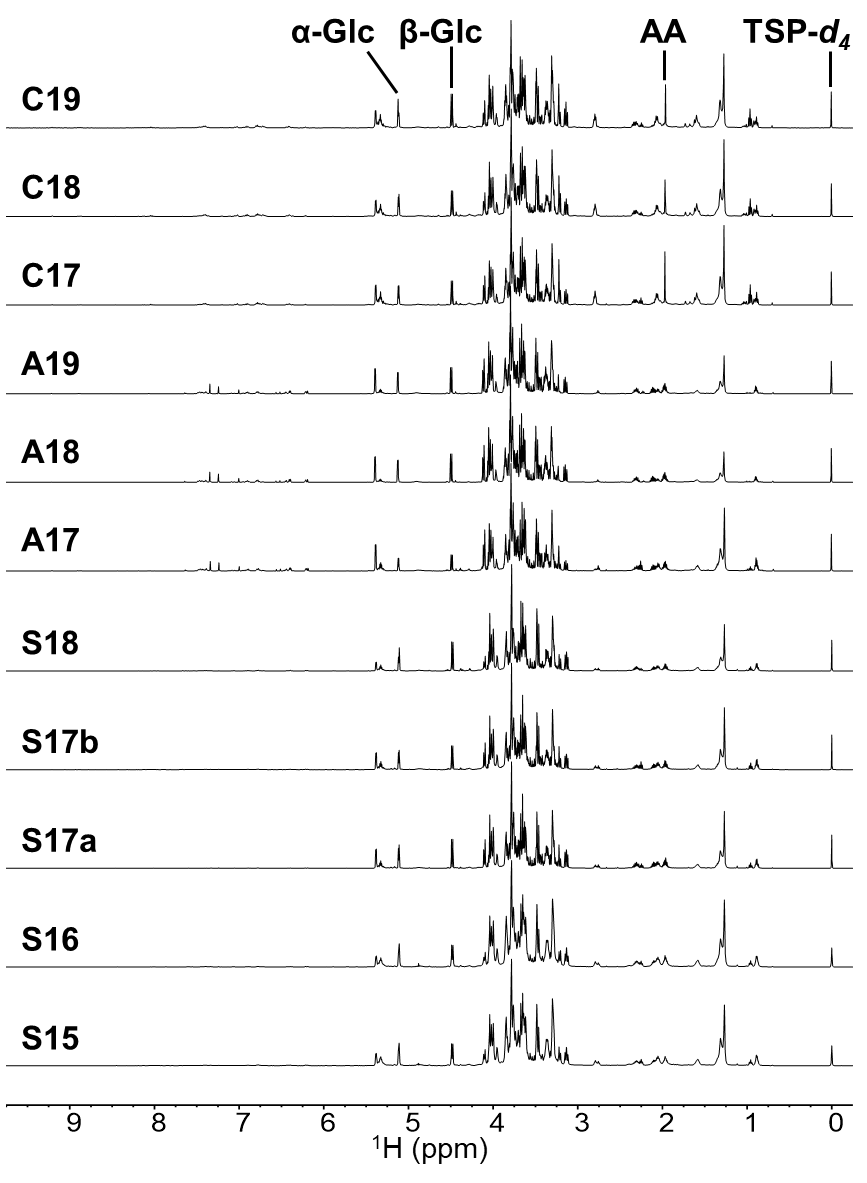


**Figure S1** The overall view of the ^1^H NMR spectrum of D_2_O extracts (a) and CD_3_OD extracts (b) of bee pollens.

**
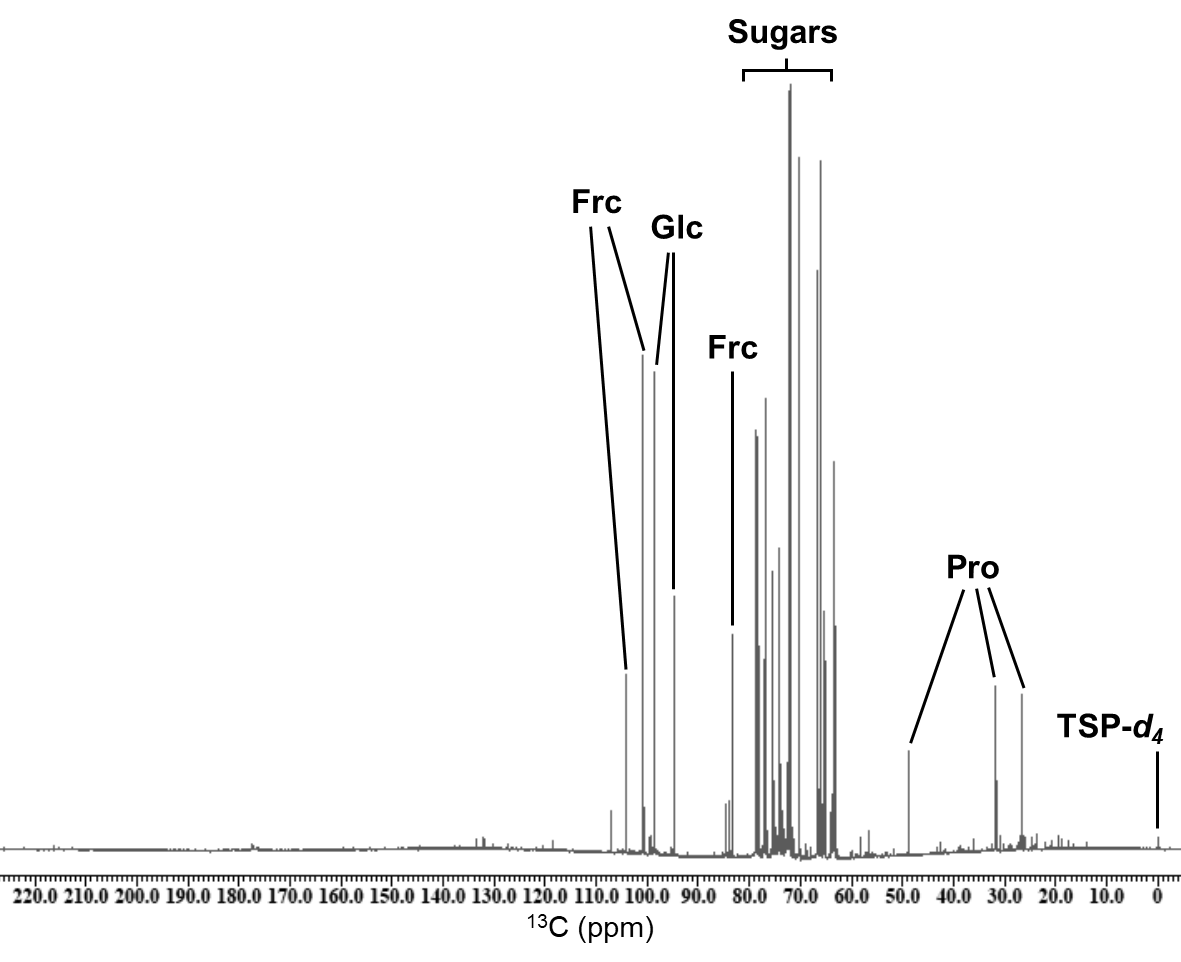
(a)**

**
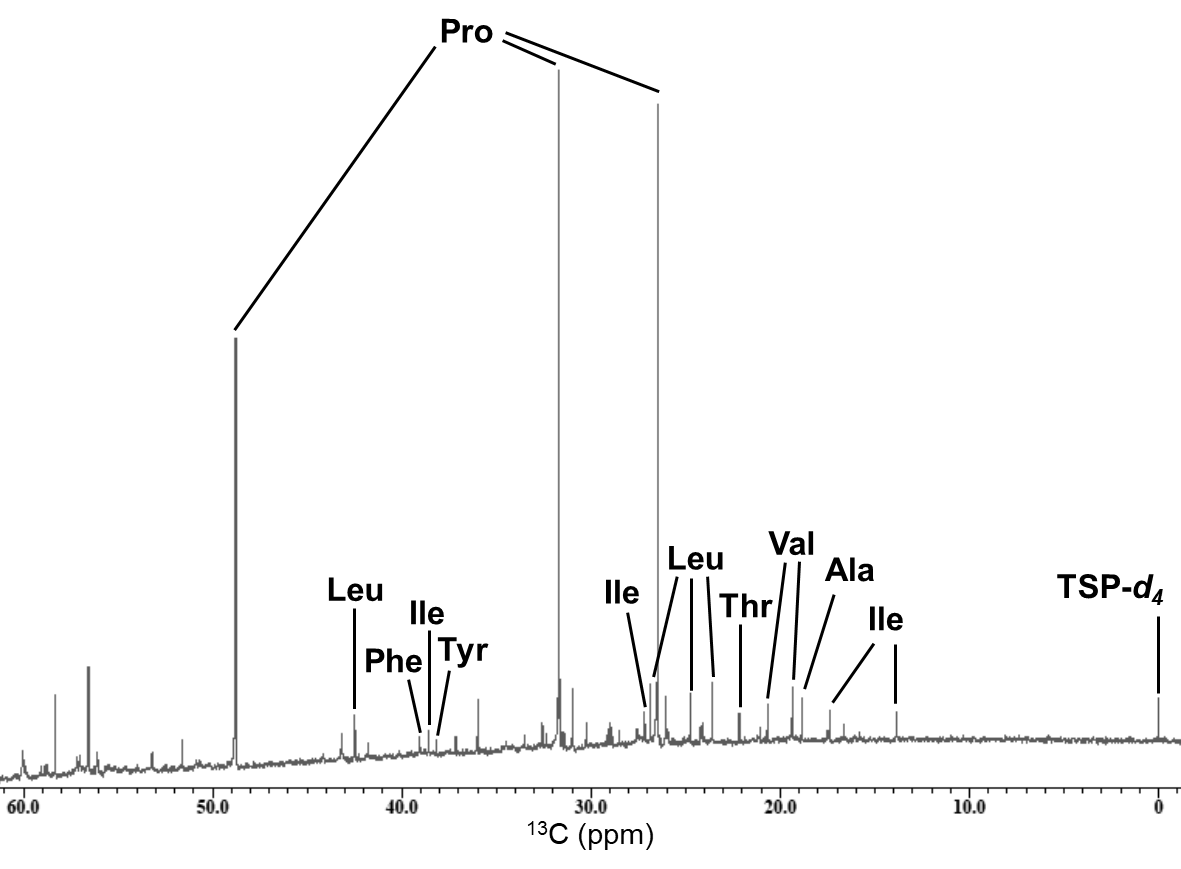
(b)**

**
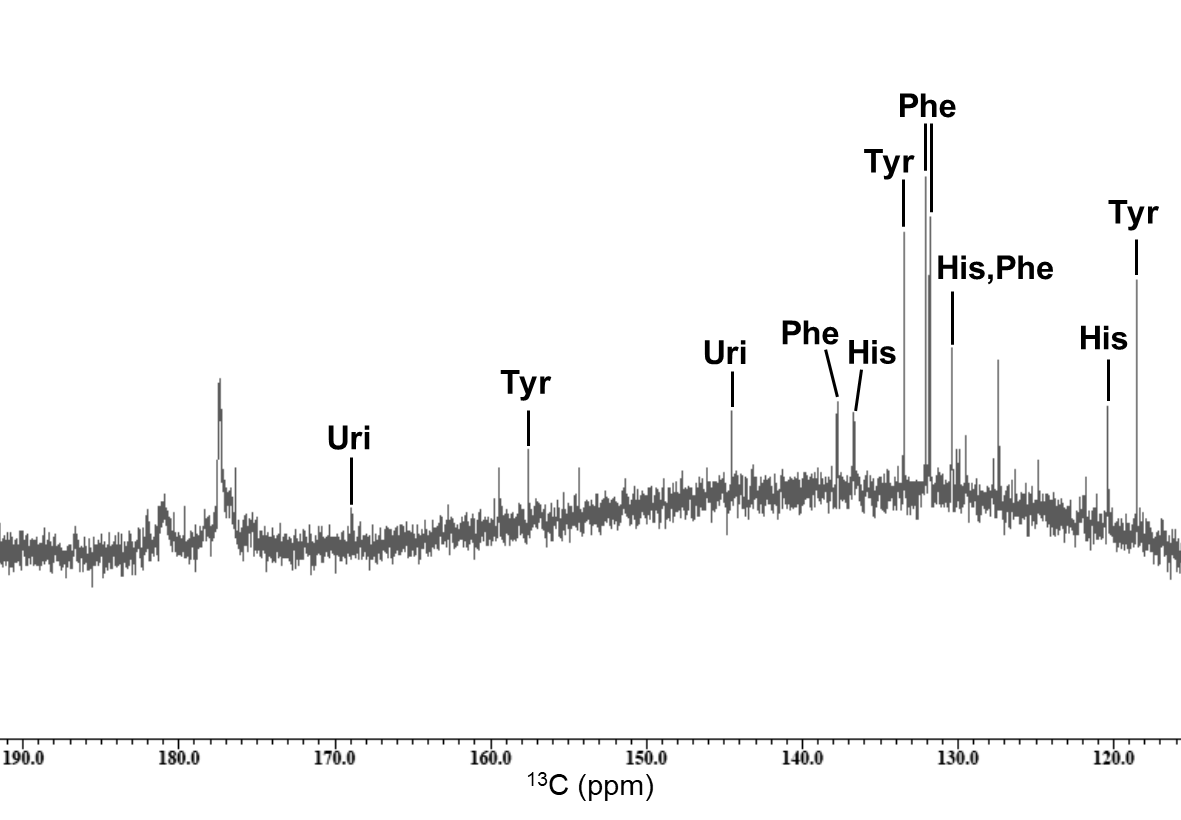
(c)**

**Figure S2** ^13^C NMR spectrum of S16 D_2_O extract of bee pollens. (a) the whole region, (b) the aliphatic region, and (c) the aromatic region.

**
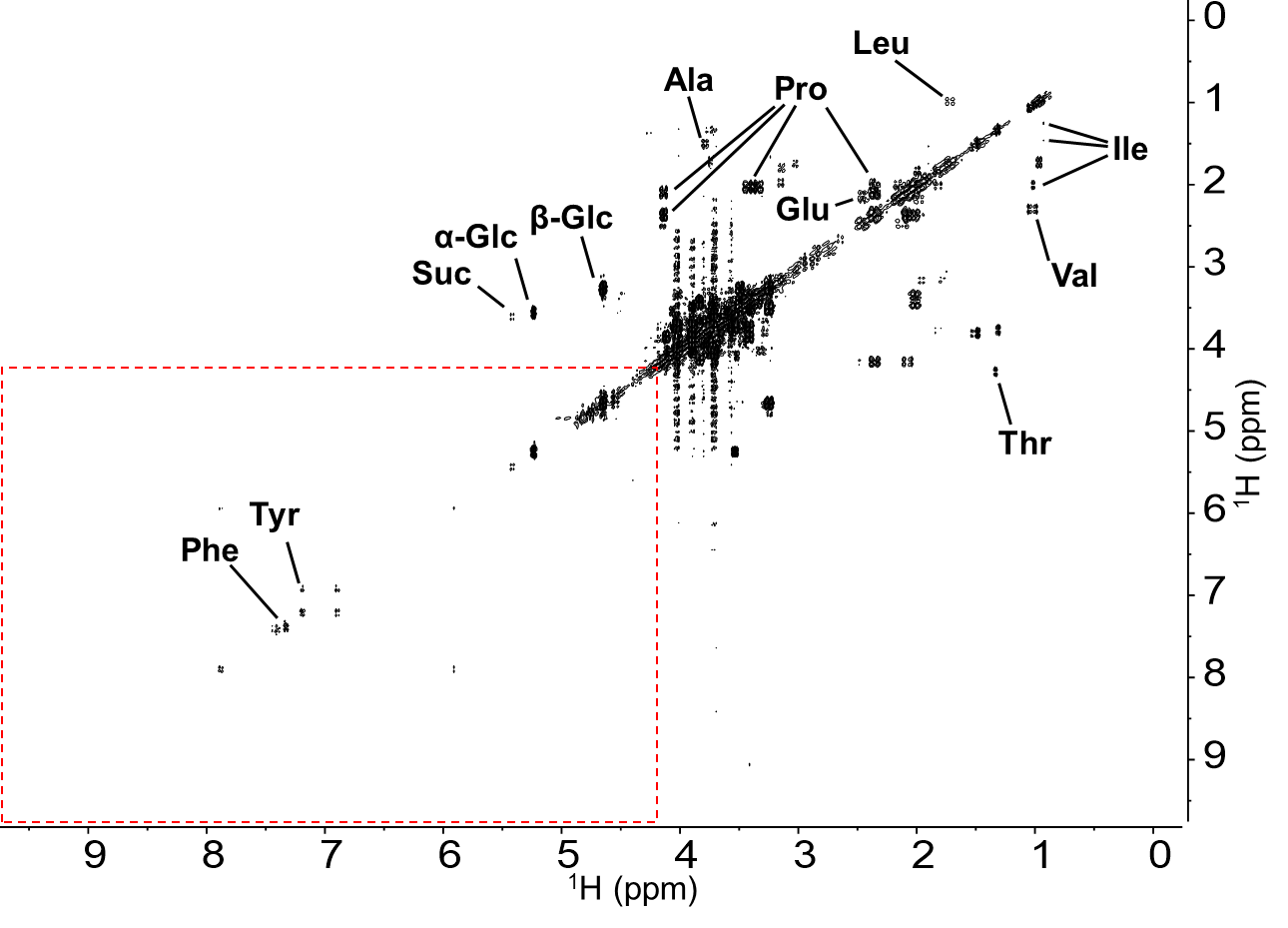
(a)**

**
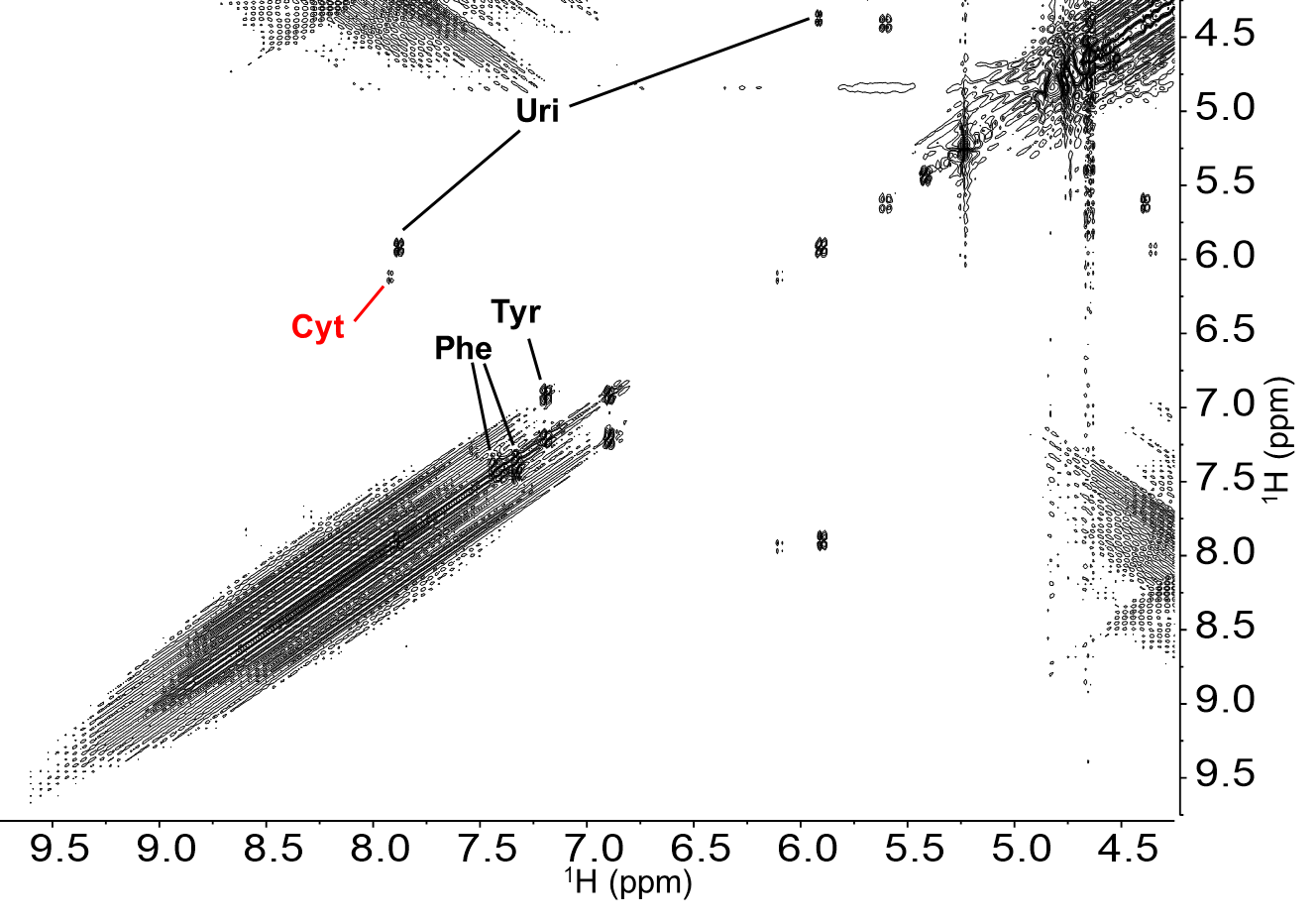
**

**(b)**

**
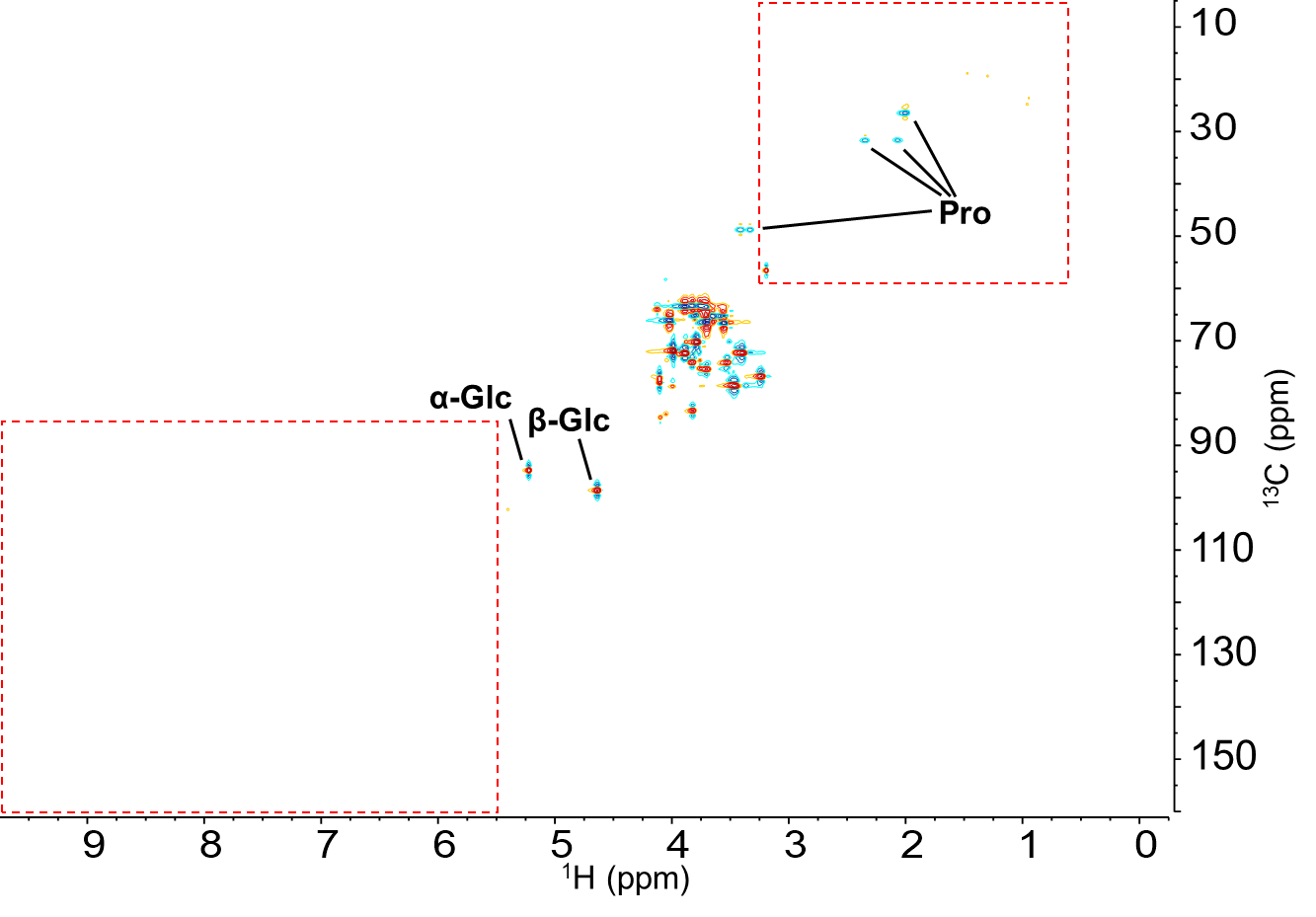
(c)**

**
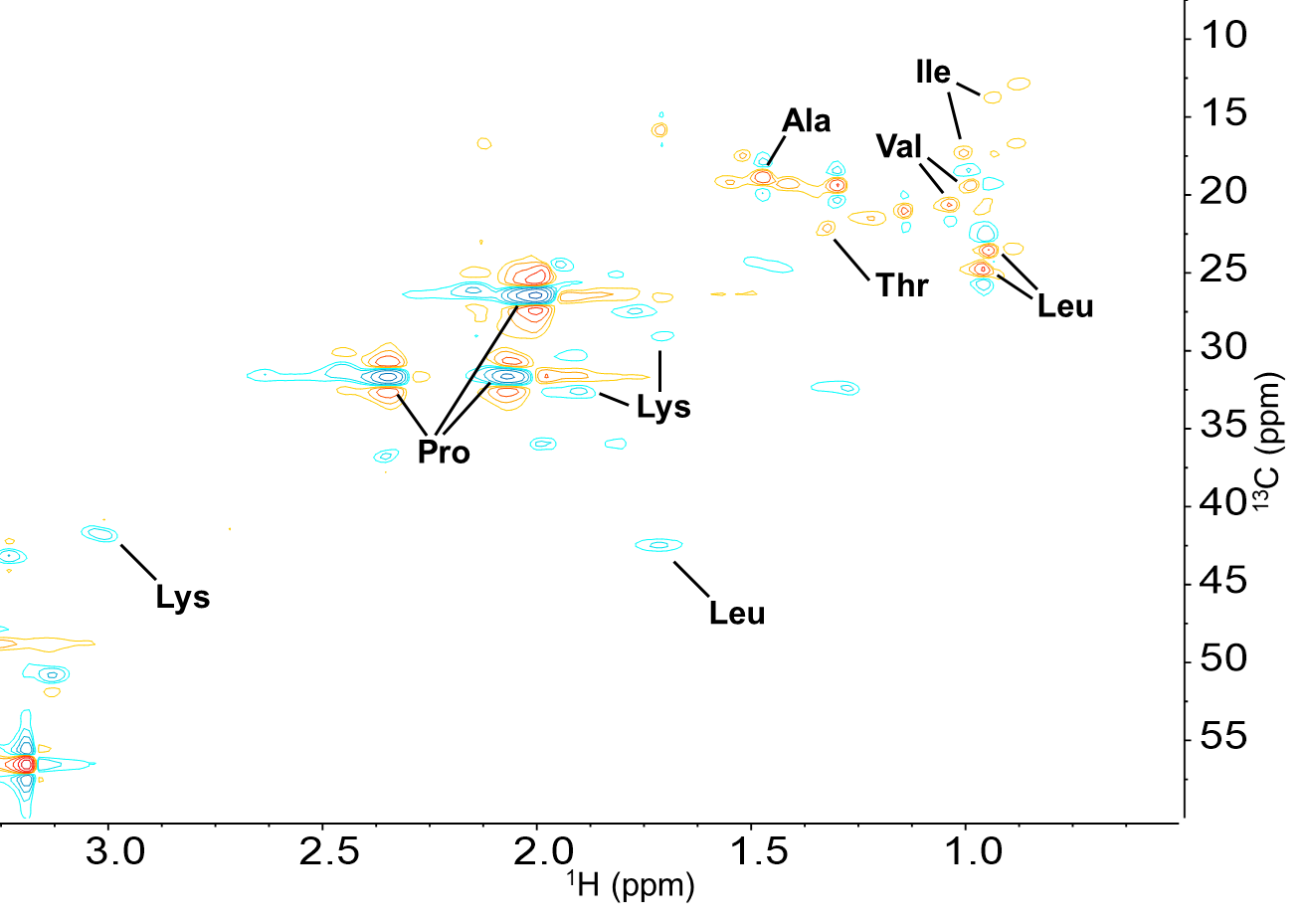
(d)**

**
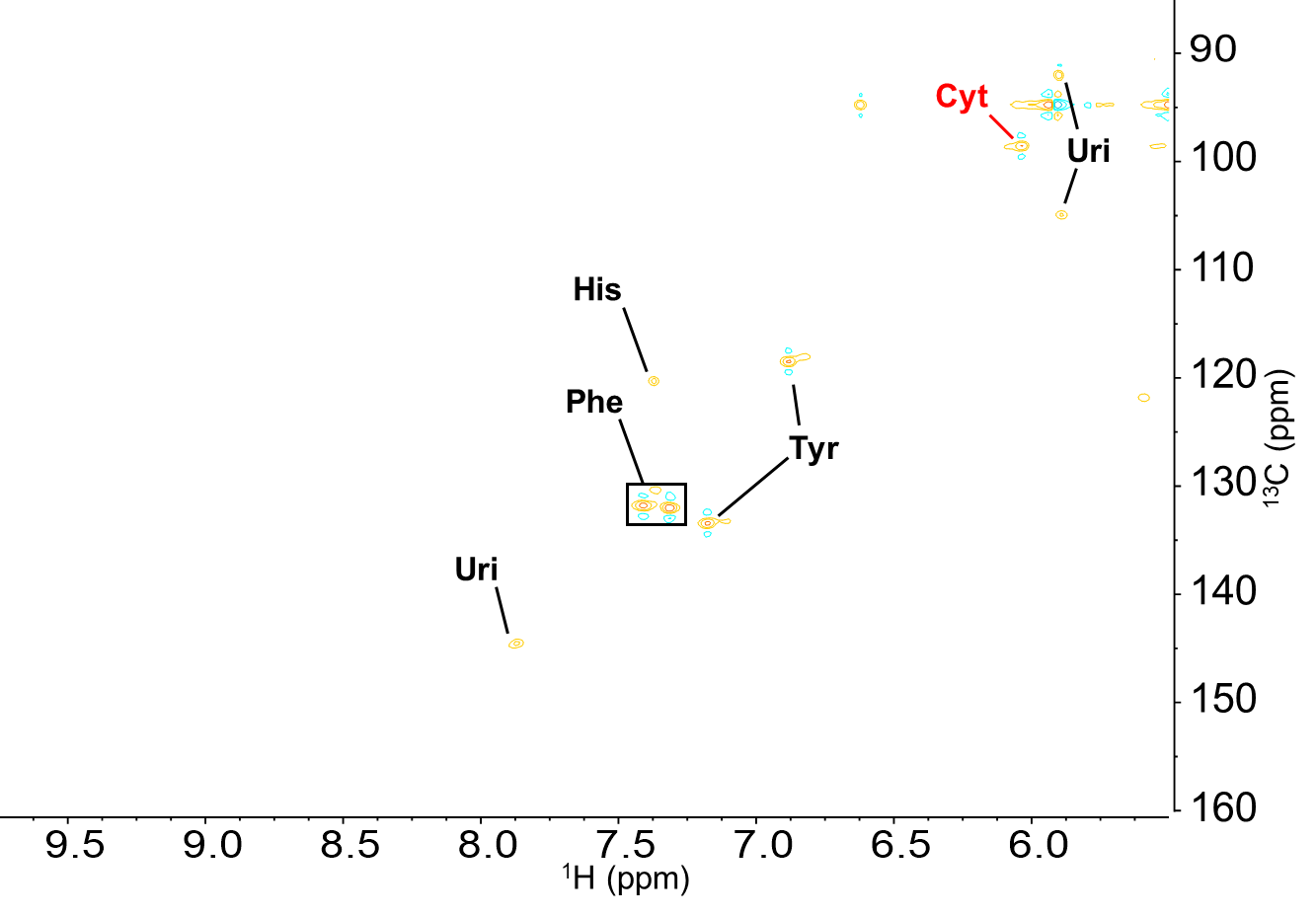
(e)**

**
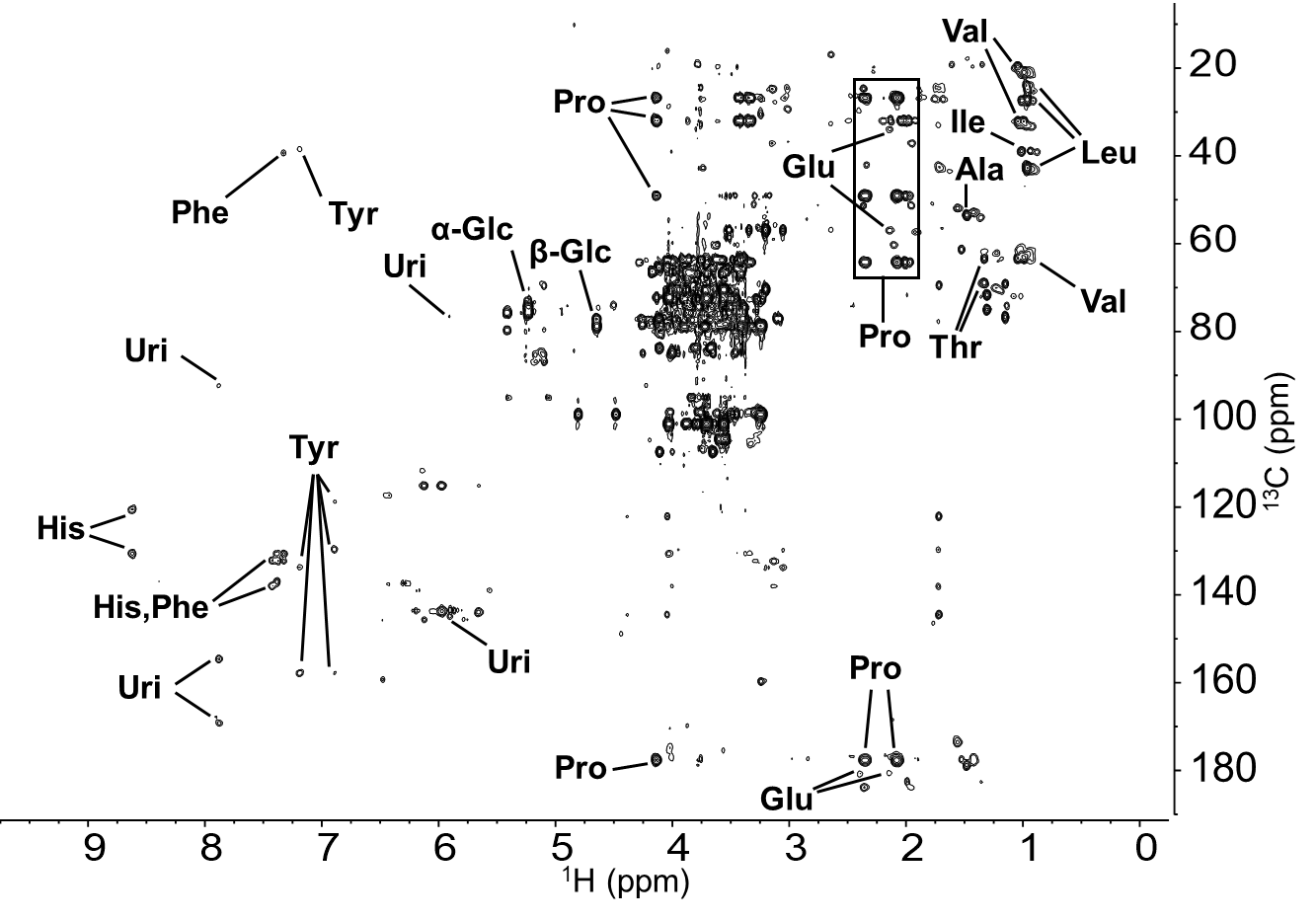
(f)**

**Figure S3** 2D NMR spectra of S16 D_2_O extract of bee pollens. (a-b) ^1^H-^1^H DQF-COSY (c-e) ^1^H-^13^C HSQC, and (f) ^1^H-^13^C HMBC spectra. (b, d, e) Zoomed spectra for the boxed regions in (a, c). Components characteristic to Spanish bee pollen samples are labeled in red.

**
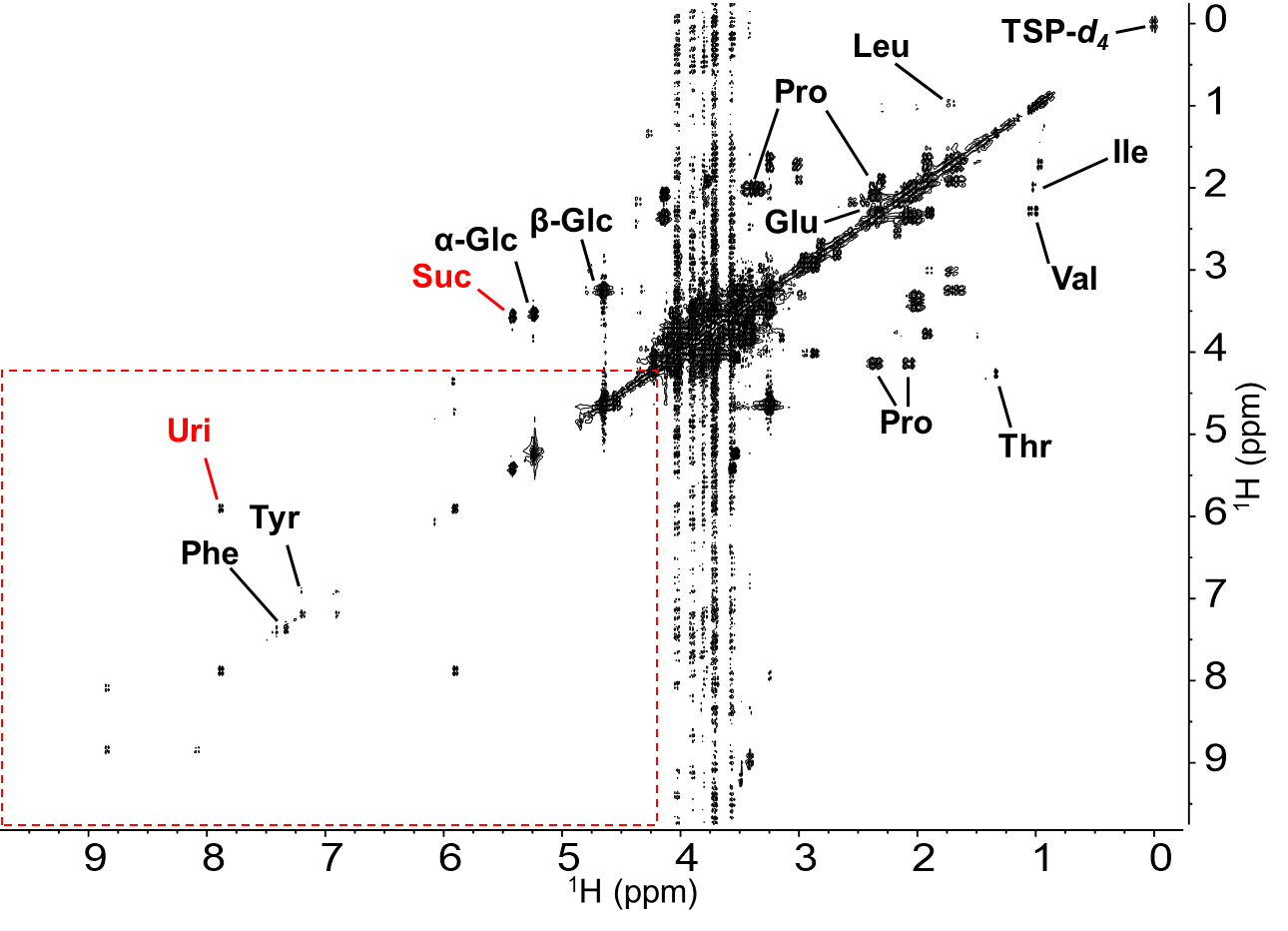
(a)**

**
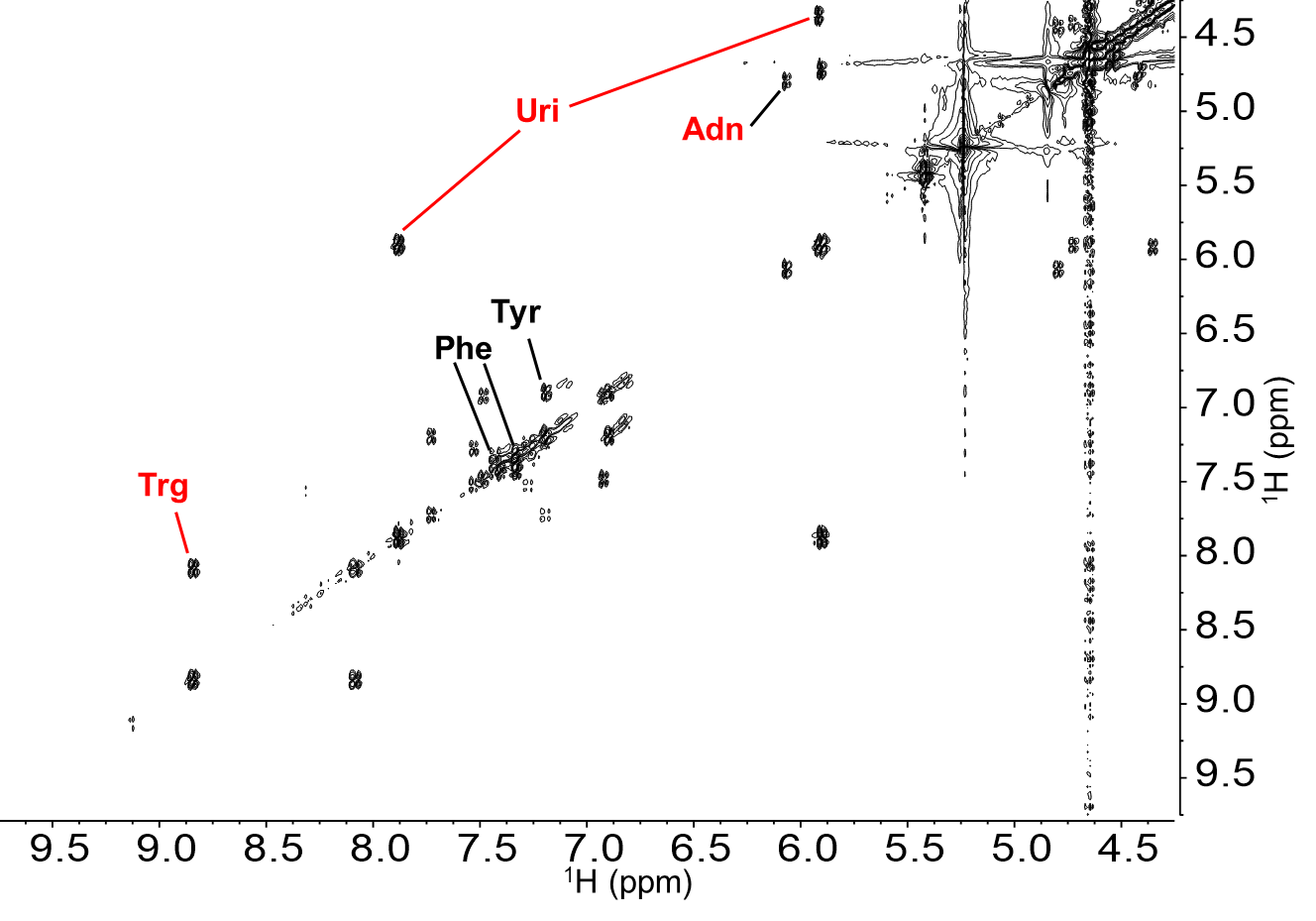
**

**(b)**

**
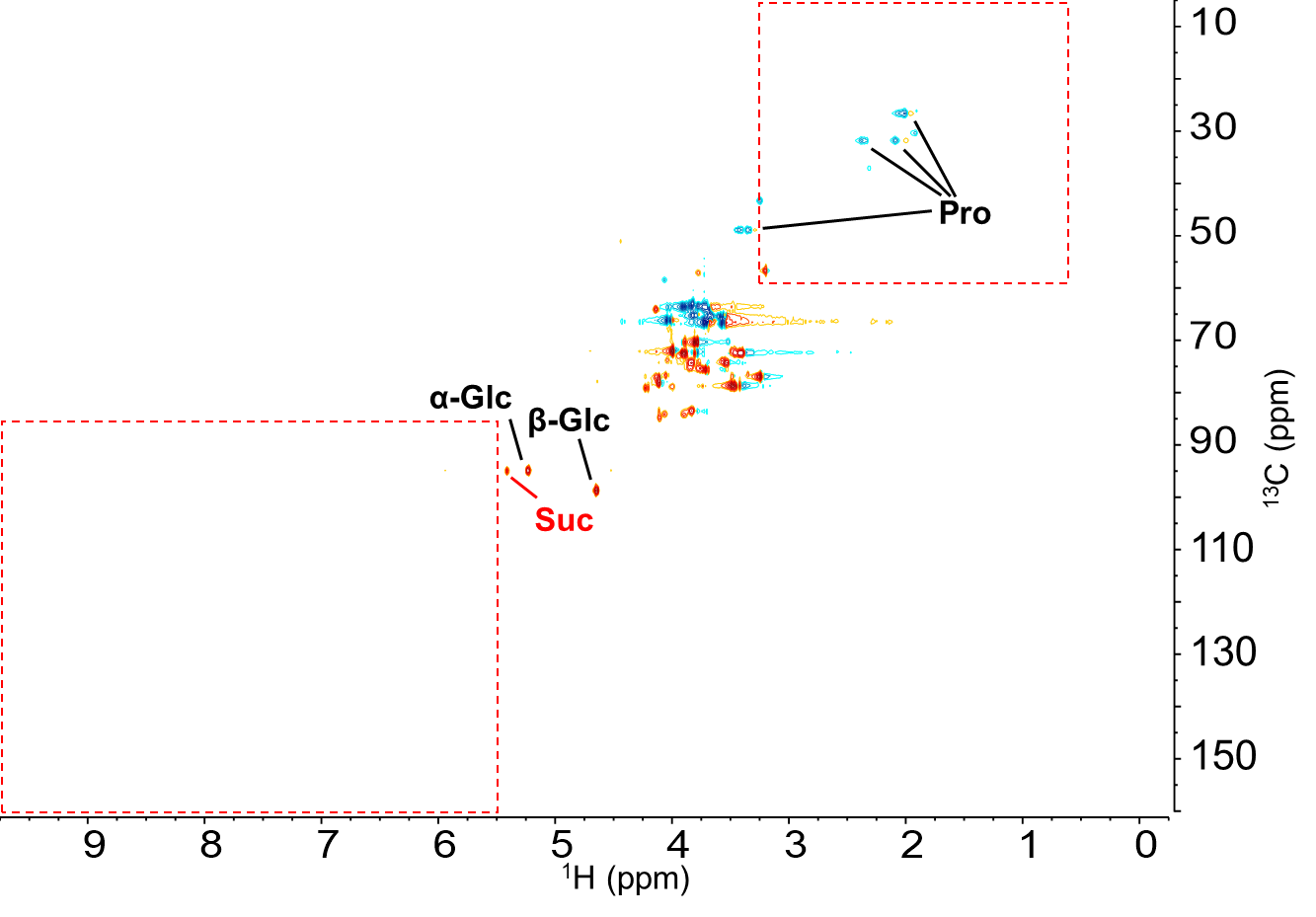
(c)**

**
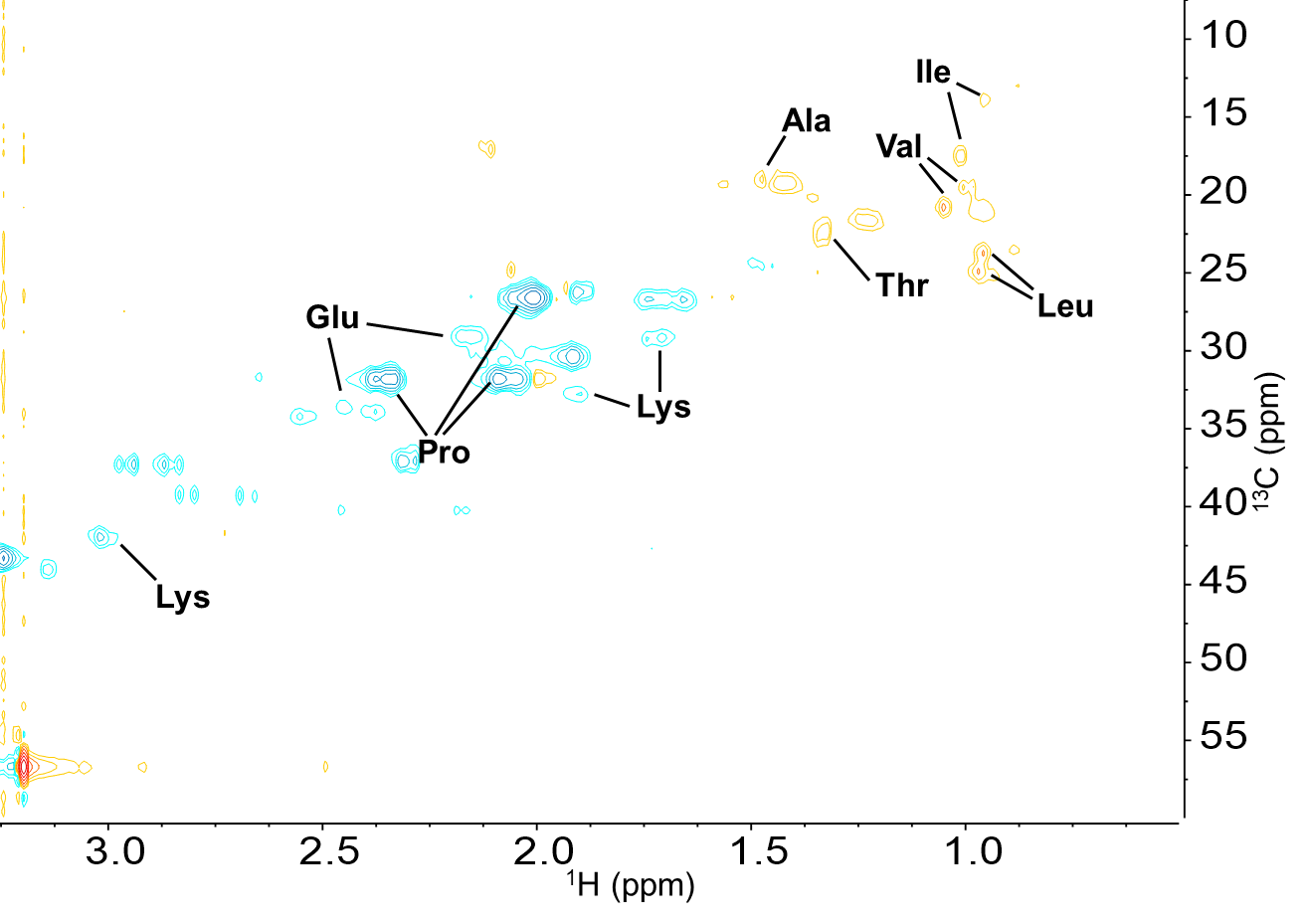
(d)**

**
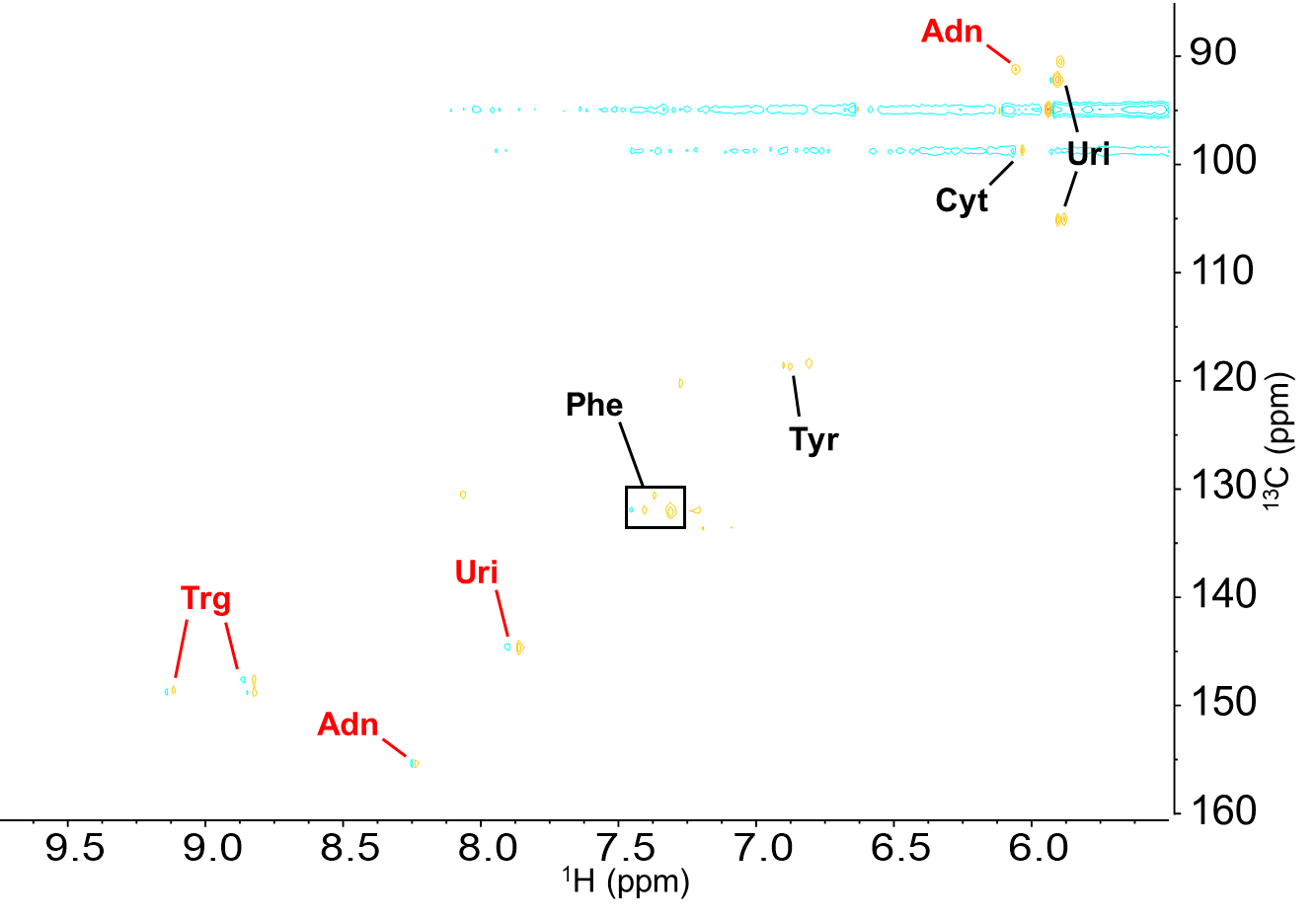
(e)**

**
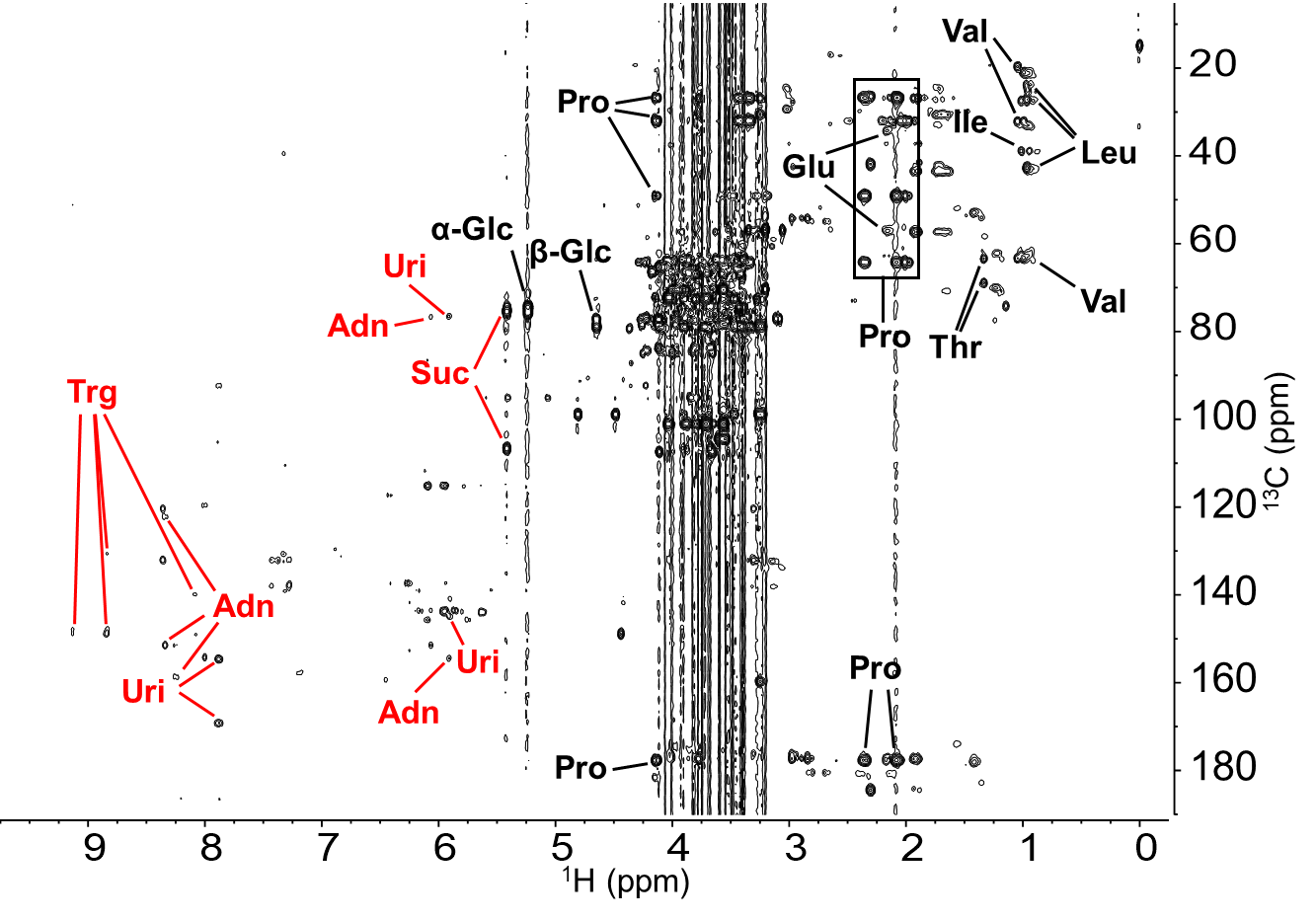
**

**(f)**

**Figure S4** 2D NMR spectra of A18 D_2_O extract of bee pollens. (a-b) ^1^H-^1^H DQF-COSY (c-e) ^1^H-^13^C HSQC, and (f) ^1^H-^13^C HMBC spectra. (b, d, e) Zoomed spectra for the boxed regions in (a, c). Components characteristic to Australian bee pollen samples are labeled in red.

**
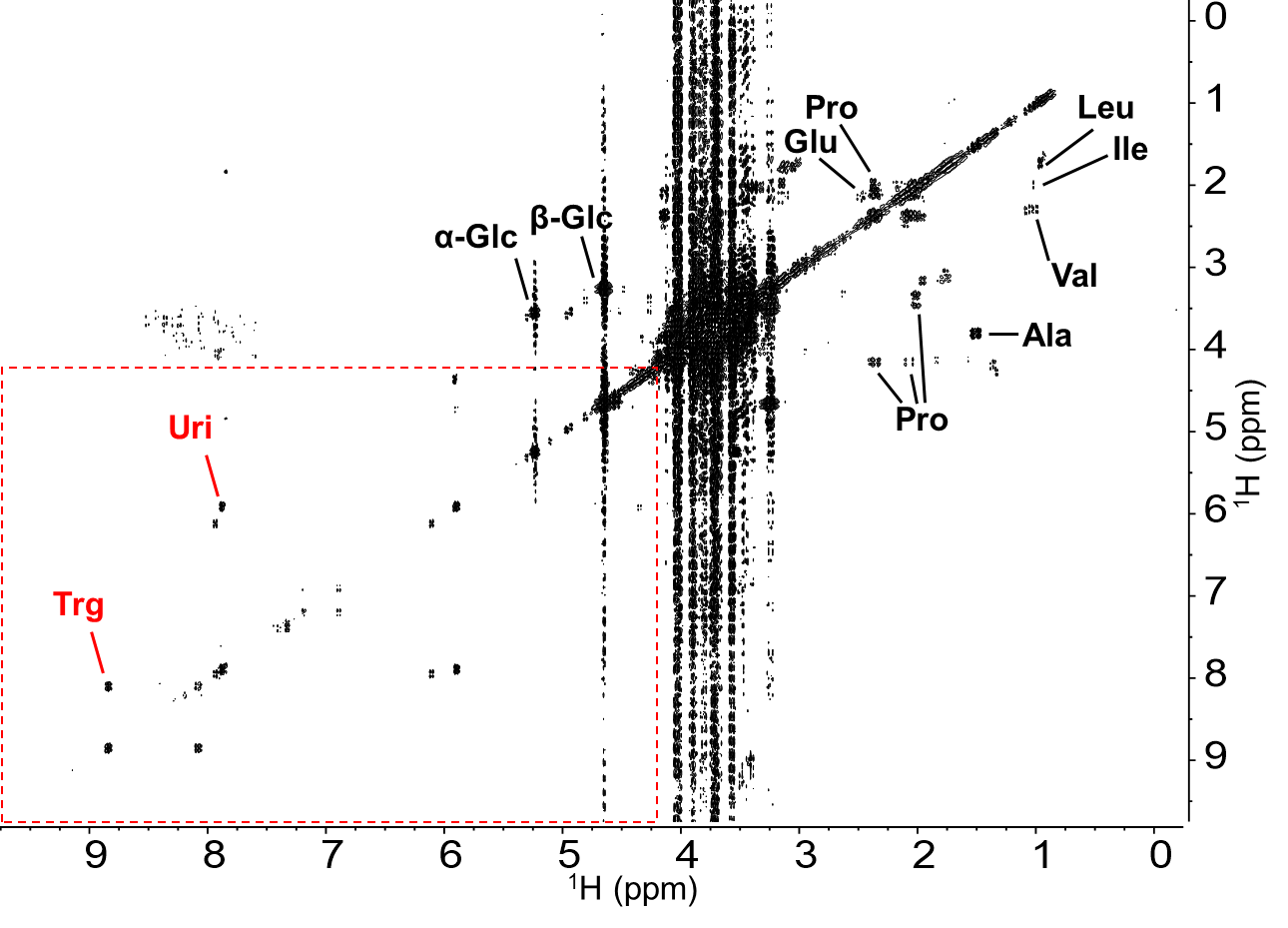
(a)**

**
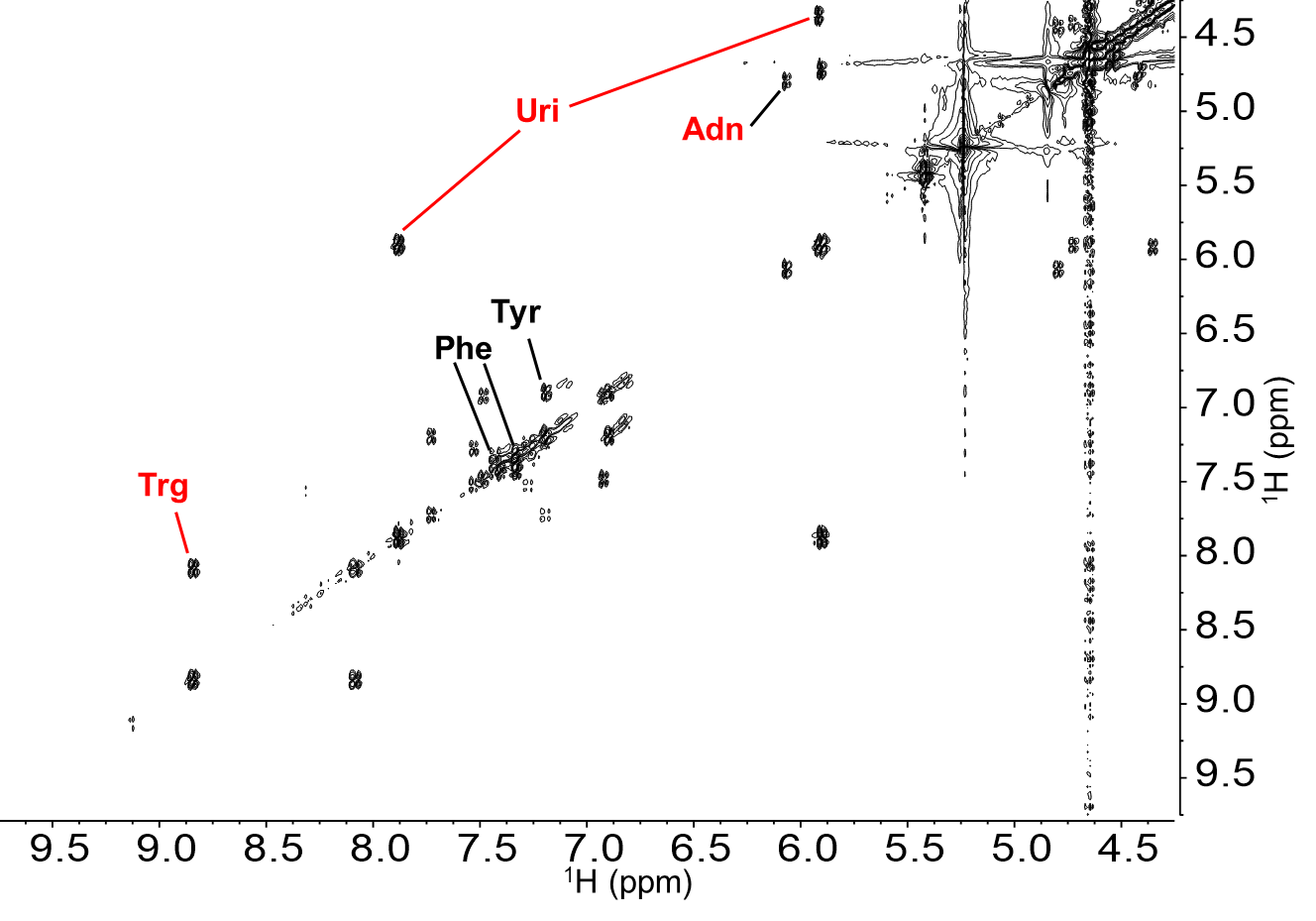
**

**(b)**

**
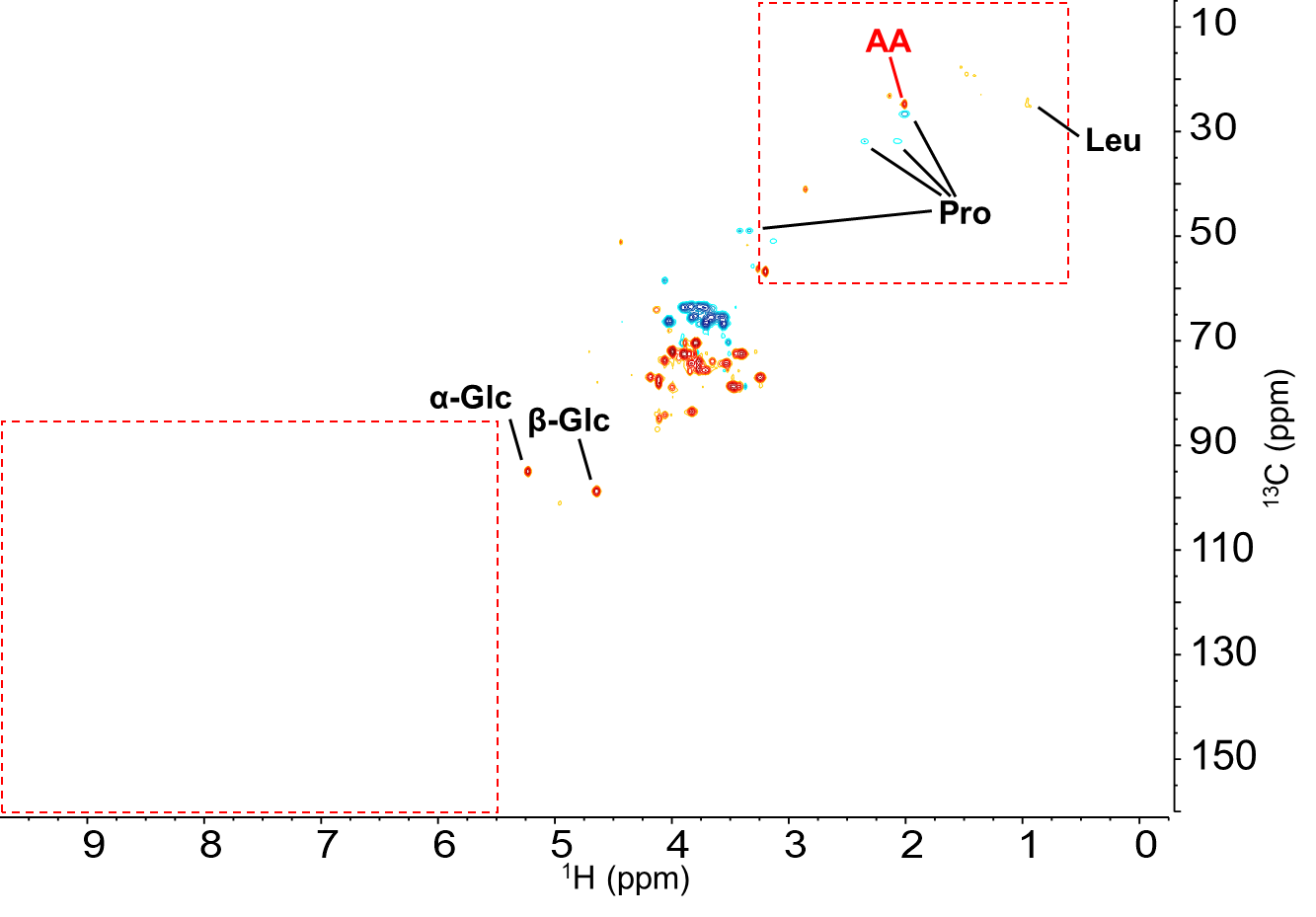
(c)**

**
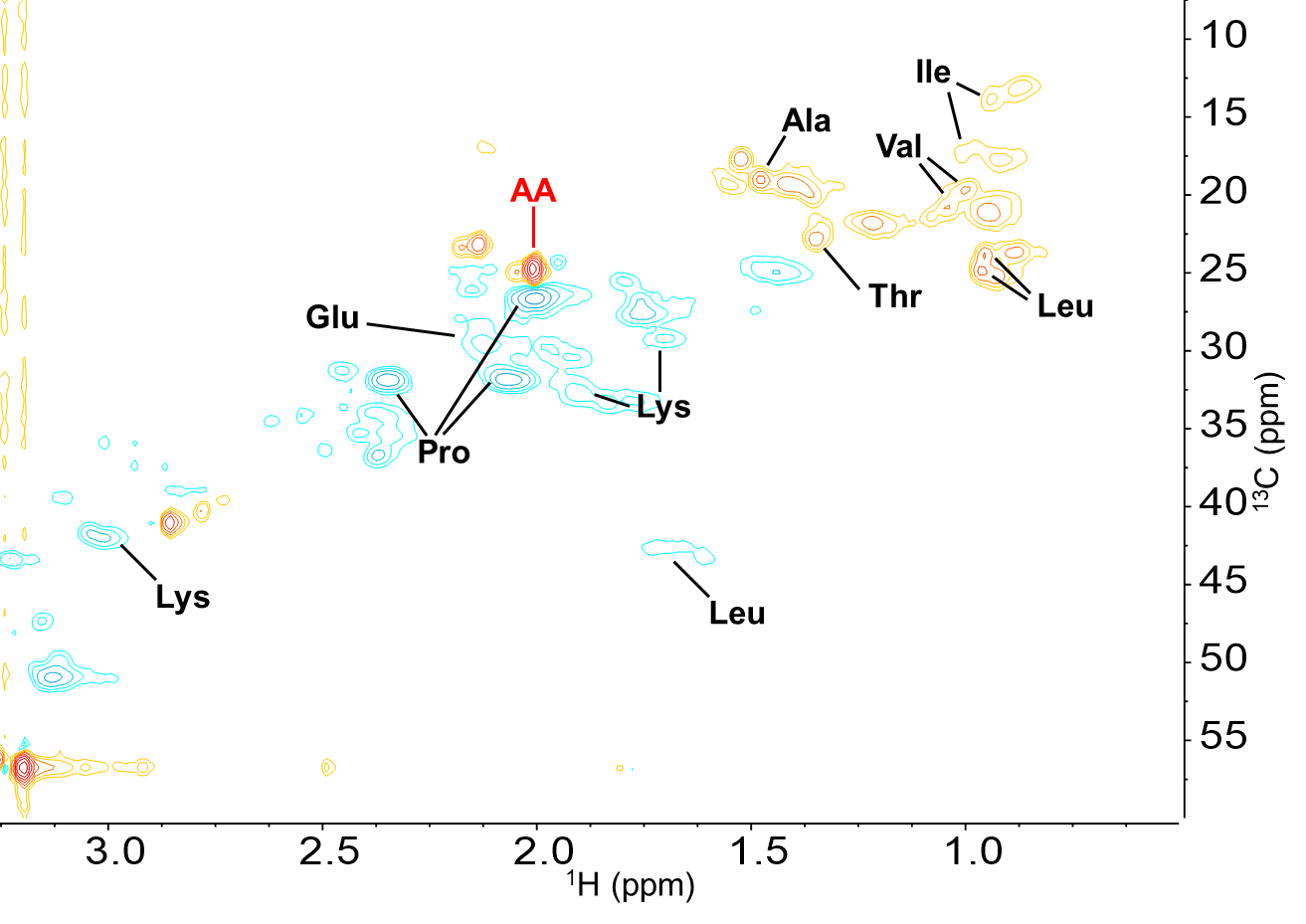
(d)**

**
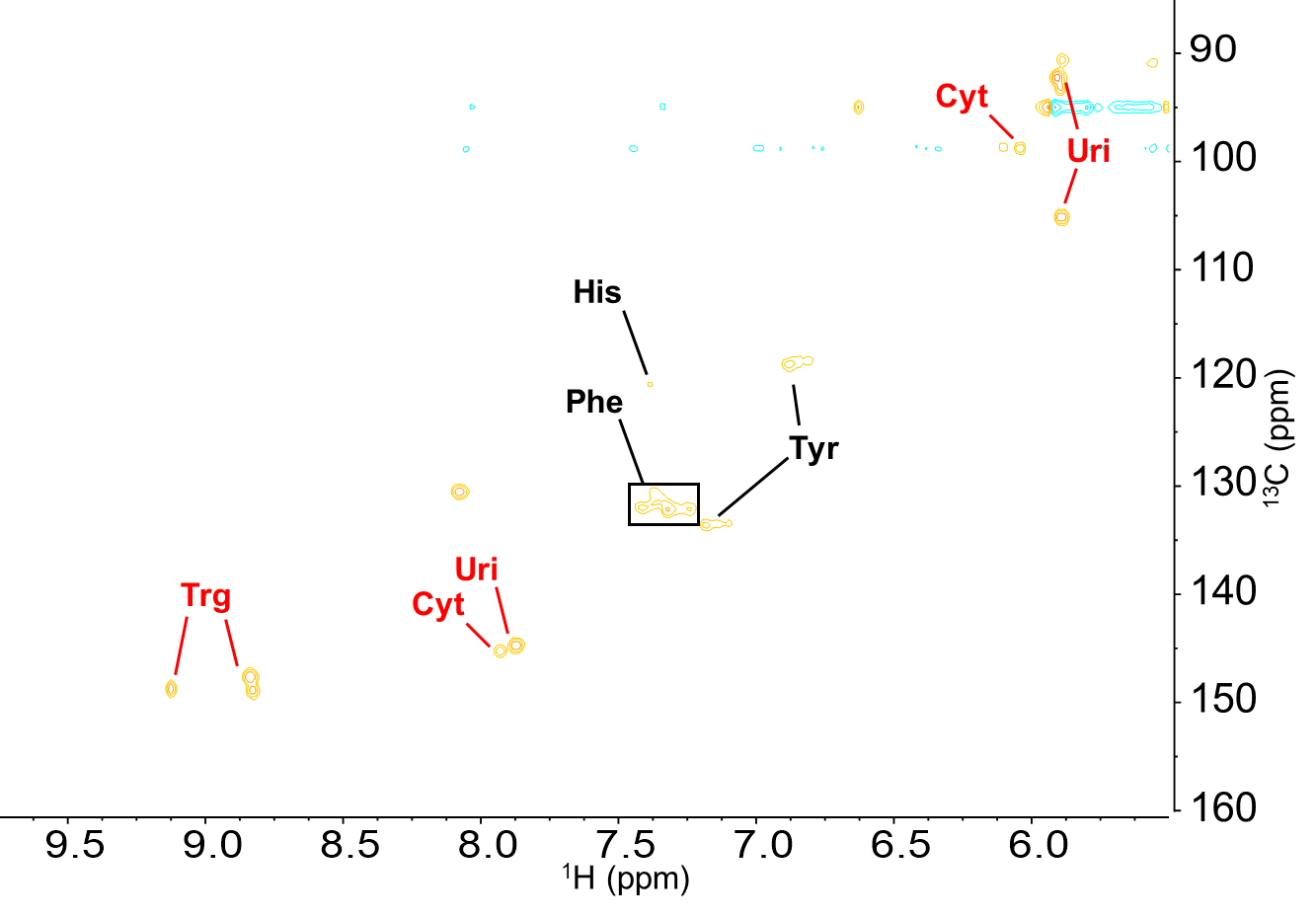
(e)**

**
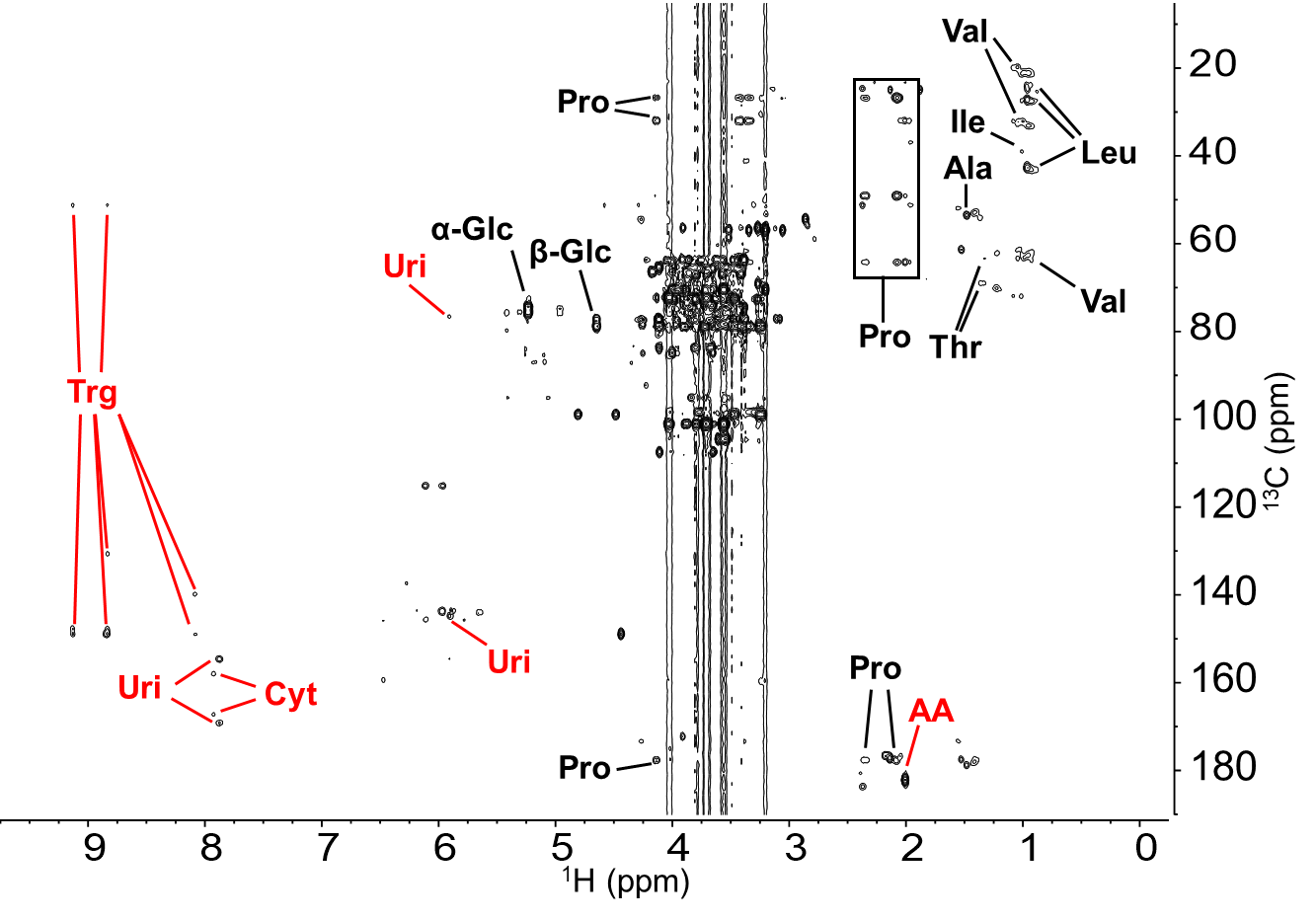
**

**(f)**

**Figure S5** 2D NMR spectra of C18 D_2_O extract of bee pollens. (a-b) ^1^H-^1^H DQF-COSY (c-e) ^1^H-^13^C HSQC, and (f) ^1^H-^13^C HMBC spectra. (b, d, e) Zoomed spectra for the boxed regions in (a, c). Components characteristic to Chinese bee pollen samples are labeled in red.
